# Supplementary material for: Caveolin-1-mediated sphingolipid oncometabolism underlies a metabolic vulnerability of prostate cancer
Source: Nat Commun. 2020 Aug 27;11:4279. doi: 10.1038/s41467-020-17645-z (PMC7453025; doi:10.1038/s41467-020-17645-z)
Supplement: Supplementary file 1 — Supplementary Information [file 41467_2020_17645_MOESM1_ESM.pdf]

Supplementary Information

Caveolin-1-Mediated Sphingolipid Oncometabolism Underlies a Metabolic Vulnerability of Prostate Cancer

Jody Vykoukal[1,7,9], Johannes F. Fahrmann[1,9], Justin R. Gregg[2], Zhe Tang[3], Spyridon Basourakos[3], Ehsan Irajizad[5], Sanghee Park[3], Guang Yang[3], Chad J. Creighton[4,7], Alia Fleury[1], Jeffrey Mayo[1], Adriana Paulucci[6], Jennifer B. Dennison[1], Eunice Murage[1], Christine B. Petterson[5], John Davis[2], Jeri Kim[3,10], Samir Hanash[1,7,10] and Timothy C. Thompson[3,10]

Departments of [1]Clinical Cancer Prevention, [2]Urology, [3]Genitourinary Medical Oncology, [4]Bioinformatics and Computational Biology, and [5]Biostatistics and [6]Genetics, and [7]McCombs Institute for the Early Detection and Treatment of Cancer, The University of Texas MD Anderson Cancer Center, 1515 Holcombe Boulevard, Houston, TX 77030, USA
[8]Dan L Duncan Comprehensive Cancer Center Division of Biostatistics, Baylor College of Medicine, One Baylor Plaza, Houston, TX 77030 USA

[9]These authors contributed equally: Jody Vykoukal, Johannes F. Fahrmann
[10]These authors jointly supervised this work: Jeri Kim, Samir Hanash, Timothy C. Thompson

Supplementary Methods

Isolation of Extracellular Vesicles by density gradient flotation. Extracellular vesicles were isolated as previously described[1]. Briefly, microvesicles were depleted from biospecimen samples by centrifugation at 2000 ×*g* for 20min followed by 16,500 ×*g* for 30min; resulting supernatant was filtered through a pre-wetted 0.22 µm vacuum filter. Microvesicle-depleted biospecimen was densified by mixing with OptiPrep iodixanol solution (Sigma D1556) to a final density of 1.16-1.30 g mL^{-1} and loaded into the bottom of a polycarbonate ultracentrifuge tube and overlaid with 0.5-2 mL aliquots of iodixanol/PBS solution in the 1.20-1.01 g mL^{-1} (35-0% wt:vol) range, proceeding from the highest to lowest density to form a single- or multi-step density fractionation gradient as needed. Ultracentrifugation was performed for at 100,000 ×*g* for 4 hrs at 8°C. Vesicles were collected from the top of the tube, proceeding downward to recover volume equal to 90% of overlaid gradient volume. Density of harvested fractions was assessed against a standard curve based on sample absorbance at 250 nm using a NanoDrop microvolume spectrophotometer (ThermoFisher Scientific, Wilmington, DE). Vesicle harvests were stored at -80°C.

Proteomic Profiling of Extracellular Vesicles. Proteomic profiling of extracellular vesicles was performed according to the following standardized workflows[1,2]. Briefly, ECV-derived protein digestion and identification by LC-MS/MS was performed using established protocols. NanoAcquity UPLC system coupled in-line with WATERS SYNAPT G2-Si mass spectrometer was used for the separation of pooled digested protein fractions. The system was equipped with a Waters Symmetry C18 nanoAcquity trap-column (180 µm × 20 mm, 5 µm) and a Waters HSS-T3 C18 nanoAcquity analytical column (75 µm × 150 mm, 1.8 µm). The column oven temperature was set at 50 °C, and the temperature of the tray compartment in the auto-sampler was set at 6 °C. LC-HDMSE data were acquired in resolution mode with SYNAPT G2-Si using Waters Masslynx (version 4.1, SCN 851). The capillary voltage was set to 2.80 kV, sampling cone voltage to 30 V, source offset to 30 V and source temperature to 100 °C. Mobility utilized high-purity N2 as the drift gas in the IMS TriWave cell. Pressures in the helium

cell, Trap cell, IMS TriWave cell and Transfer cell were 4.50 mbar, 2.47×10^{-2}, 2.90, and 2.53×10^{-3} mbar, respectively. IMS wave velocity was 600 m s^{-1}, helium cell DC 50 V, Trap DC bias 45 V, IMS TriWave DC bias V and IMS wave delay 1000 µs. The mass spectrometer was operated in V-mode with a typical resolving power of at least 20,000. All analyses were performed using positive mode ESI using a NanoLockSpray source. The lock mass channel was sampled every 60 s. The mass spectrometer was calibrated with a [Glu1] fibrinopeptide solution (300 fmol µL^{-1}) delivered through the reference sprayer of the NanoLockSpray source. Accurate mass LC-HDMSE data were collected in an alternating, low energy (MS) and high energy (MSE) mode of acquisition with mass scan range from m z^{-1} 50 to 1800. The spectral acquisition time in each mode was 1.0 s with a 0.1-s inter-scan delay. In low energy HDMS mode, data were collected at constant collision energy of 2 eV in both Trap cell and Transfer cell. In high-energy HDMSE mode, the collision energy was ramped from 25 to 55 eV in the Transfer cell only. The RF applied to the quadrupole mass analyzer was adjusted such that ions from m z^{-1} 300 to 2000 were efficiently transmitted, ensuring that any ions observed in the LC-HDMSE data < $m\ z^{-1}$ of 300 arose from dissociations in the Transfer collision cell. The acquired LC-HDMSE data were processed and searched against protein knowledge database (Uniprot) through ProteinLynx Global Server (PLGS, Waters Company) with 4% FDR.

Metabolomics Analyses. **Sample Extraction.** Following transfections, cell lysates were washed 2x with pre-chilled 0.9% NaCl followed by addition of 2.5 mL of pre-chilled 3:1 isopropanol:ultrapure water. Cells were scraped using a 25 cm Cell Scraper (Sarstedt) in extraction solvent and transferred to a 15 mL conical tube (Eppendorf). Samples were briefly vortexed followed by centrifugation at 4°C for 10 min at 2,000 ×*g*. Thereafter, 1.2mL of metabolite extracts were transferred to 1.5mL Eppendorf tubes and stored in -20°C until metabolomic analysis. **Primary Metabolites and Biogenic Amines.** Plasma metabolites were extracted from pre-aliquoted EDTA plasma (10 µL) with 30µL of LCMS grade methanol (ThermoFisher) in a 96-well microplate (Eppendorf). Plates were heat sealed, vortexed for 5 min at 750 rpm, and centrifuged at 2000 × g for 10 minutes at room temperature. The supernatant (10 µL) was carefully transferred to a 96-well plate, leaving

behind the precipitated protein. The supernatant was further diluted with 10 µL of 100 mM ammonium formate, pH3. For Hydrophilic Interaction Liquid Chromatography (HILIC) analysis, the samples were diluted with 60 µL LCMS grade acetonitrile (ThermoFisher), whereas samples for C18 analysis were diluted with 60 µL water (GenPure ultrapure water system, ThermoFisher). Each sample solution was transferred to 384-well microplate (Eppendorf) for LCMS analysis. For conditioned media, frozen samples were thawed on ice and 30µl transferred to a 96-well microplate (Eppendorf). Aliquots were diluted with an additional 30 µL of 100 mM ammonium formate. Microplates were heat sealed, vortexed for 5min at 1500rpm, and centrifuged at 2000 ×*g* for 10minutes at room temperature. For Hydrophilic Interaction Liquid Chromatography (HILIC) analysis, the 25µL of sample was transferred to a new 96 well microplate containing 75µL acetonitrile, whereas samples for C18 analysis were transferred to a new 96-well microplate containing 75µL water (GenPure ultrapure water system, ThermoFisher). Each sample solution was transferred to 384-well microplate (Eppendorf) for LCMS analysis. Cell lysate supernatant, 100 µL (3:1 isopropanol:ultrapure water) was aliquoted into two 96-well plates (Eppendorf) and evaporated to dryness under vacuum. The samples were then reconstituted as follows: for the HILIC assays, the dried samples were dissolved in 65 µL of ACN (ThermoFisher): 100 mM Ammonium Formate pH3 (9:1) whereas for the C18 reverse phase assays, the dried samples were dissolved in 65 µL of H2O: 100 mM Ammonium Formate pH3 (9:1). The samples were spun down to remove any insoluble materials and then transferred to a 384-well plate for high throughput mass analysis using LCMS. **Complex Lipids**. Pre-aliquoted EDTA plasma samples (10 µL) were extracted with 30 µL of LCMS grade 2-propanol (ThermoFisher) in a 96-well microplate (Eppendorf). Plates were heat sealed, vortexed for 5 min at 750 rpm, and centrifuged at 2000 ×*g* for 10 minutes at room temperature. The supernatant (10µL) was carefully transferred to a 96-well plate, leaving behind the precipitated protein. The supernatant was further diluted with 90 µL of 1:3:2 100mM ammonium formate, pH3 (ThermoFisher): acetonitrile: 2-propanol and transferred to a 384-well microplate (Eppendorf) for lipids analysis using LCMS. For cell lysates, in a 96 well plate, 10µL (3:1 isopropanol:ultrapure water) of the cell lysates supernatant was diluted with 90µL of 1:3:2 100mM ammonium formate, pH3: acetonitrile: 2-propanol (ThermoFisher) and transferred to a 384-well

microplate (Eppendorf) for analysis by LC-MS. **Untargeted Analysis of Primary Metabolites and Biogenic Amines.** Untargeted metabolomics analysis was conducted on Waters Acquity™ UPLC system with 2D column regeneration configuration (I-class and H-class) coupled to a Xevo G2-XS quadrupole time-of-flight (qTOF) mass spectrometer. Chromatographic separation was performed using HILIC (Acquity™ UPLC BEH amide, 100 Å, 1.7 µm 2.1× 100mm, , Waters Corporation, Milford, U.S.A) and C18 (Acquity™ UPLC HSS T3, 100 Å, 1.8 µm, 2.1×100mm, Water Corporation, Milford, U.S.A) columns at 45°C. Quaternary solvent system mobile phases were (A) 0.1% formic acid in water, (B) 0.1% formic acid in acetonitrile and (D) 100mM ammonium formate, pH 3. Samples were separated using the following gradient profile: for the HILIC separation a starting gradient of 95% B and 5% D was increase linearly to 70% A, 25% B and 5% D over a 5min period at 0.4 mL min^{-1} flow rate, followed by 1 min isocratic gradient at 100 % A at 0.4 mL min^{-1} flow rate. For C18 separation, a chromatography gradient of was as follows: starting conditions, 100% A, with linear increase to final conditions of 5% A, 95% B followed by isocratic gradient at 95% B, 5% D for 1 min. Binary pump was used for column regeneration and equilibration. The solvent system mobile phases were (A1) 100mM ammonium formate, pH 3, (A2) 0.1 % formic in 2-propanol and (B1) 0.1 % formic acid in acetonitrile. The HILIC column was stripped using 90% A2 for 5 min followed by 2 min equilibration using 100% B1 at 0.3 mL min^{-1} flowrate. Reverse phase C18 column regeneration was performed using 95% A1, 5% B1 for 2 min followed by column equilibration using 5% A1, 95% B1 for 5 min. **Untargeted Analysis of Complex Lipids.** For the lipidomic assay, untargeted metabolomics analysis was conducted on a Waters Acquity™ UPLC system coupled to a Xevo G2-XS quadrupole time-of-flight (qTOF) mass spectrometer. Chromatographic separation was performed using a C18 (Acquity™ UPLC HSS T3, 100 Å, 1.8 µm, 2.1×100mm, Water Corporation, Milford, USA) column at 55°C. The mobile phases were (A) water, (B) Acetonitrile, (C) 2-propanol and (D) 500mM ammonium formate, pH 3. A starting elution gradient of 20% A, 30% B, 49% C and 1% D was increased linearly to 10% B, 89% C and 1 % D for 5.5 min, followed by isocratic elution at 10% B, 89% C and 1% D for 1.5 min and column equilibration with initial conditions for 1min.

Mass Spectrometry Data Acquisition. Mass spectrometry data was acquired using sensitivity mode in positive and negative electrospray ionization mode within 50-1200 Da range for primary metabolites and 100-2000 Da for complex lipids. For the electrospray acquisition, the capillary voltage was set at 1.5 kV (positive), 3.0 kV (negative), sample cone voltage 30V, source temperature at 120° C, cone gas flow 50 L h^{-1} and desolvation gas flow rate of 800 L h^{-1} with scan time of 0.5 sec in continuum mode. Leucine Enkephalin; 556.2771 Da (positive) and 554.2615 Da (negative) was used for lockspray correction and scans were performed at 0.5 min. The injection volume for each sample was 3 µL, unless otherwise specified. The acquisition was carried out with instrument auto gain control to optimize instrument sensitivity over the samples acquisition time.

Mass Spectrometry Data Processing. LC-MS and LC-MSe data were processed using Progenesis QI (Nonlinear, Waters) and values were reported as area units. Annotations were determined by matching accurate mass and retention times using customized libraries created from authentic standards and/or by matching experimental tandem mass spectrometry data against the NIST MSMS or HMDB v3 theoretical fragmentations.

Data Normalization. To correct for injection order drift, each feature was normalized using data from repeat injections of quality control samples collected every 10 injections throughout the run sequence. Measurement data were smoothed by Locally Weighted Scatterplot Smoothing (LOESS) signal correction (QC-RLSC) as previously described[3]. Feature values between quality control samples were interpolated by a cubic spline. Metabolite values were rescaled by using the overall median of the historical quality control peak areas across all samples. Only detected features exhibiting a relative standard deviation (RSD) less than 30 in either the historical or pooled quality controls samples were considered for further statistical analysis. To reduce data matrix complexity, annotated features with multiple adducts or acquisition mode repeats were collapsed to one representative unique feature. Features were selected based on replicate precision (RSD<30), highest intensity and best isotope similarity matching to theoretical isotope distributions. Values are reports as ratios relative to the historical

quality control reference samples that is included in every analytical run (plasma/conditioned media) or adjusted area units (lysates).

Statistical Analyses. In order to find the cut-off point for the covariate that gives the largest difference between individuals in the two already defined groups, we used the method that has been described in Contal and O'Quigley (1). Using log rank statistic-based on the groups defined by cut-off we have:

$$S_k = \sum_{i=1}^{D}[d_i^+ - d_i \frac{r_i^+}{r_i}]$$

Where D is the total number of distinct events (disease progression (DP)), d_i is the total number of DP at each event time (t_i), d_i^+ is the total number of DP when the Cav-1-sphingolipid signature value is bigger than the cut-off point. r_i and r_i^+ also define as the total number at risk for all Cav-1-sphingolipid signature values and Cav-1-sphingolipid signature values larger than cut-off point respectively. We calculated S_k for all possible cut point in Cav-1-sphingolipid signature column and the estimated cut point is the value that yields the maximum S_k. In our analysis, the maximum value of S_k is at the top 16.4% of Cav-1-sphingolipid signature values. In another word, top 16.4% of Cav-1-sphingolipid signature values are in the high-risk group and other 83.6% are in the low-risk group.
In order to calculate the *p*-value of this test we used the following formula and it gives the value of 0.009. It suggests that the Cav-1-sphingolipid signature level highly relates to progression free survival.

$$p\text{-value} \approx 2\exp(-2Q^2)$$

where:

$$Q = \frac{\max|S_k|}{s\sqrt{D-1}}$$

and

$$s^2 = \frac{1}{D-1}\sum_{i=1}^{D}\{1 - \sum_{j=1}^{i}\frac{1}{D-j+1}\}^2$$

Supplementary Table 1. Metabolite features identified in the MDACC discovery cohort.

MDACC Discovery Cohort

Accepted Compound ID	Domain	AUC[1]	*p*-value[#]	AUC[2]	*p*-value[#]	AUC[3]	*p*-value[#]
1-Methylhistidine	Amino Acid or Amide	0.41	0.381	0.50	1.000	0.52	0.867
1-METHYLNICOTINAMIDE	Amino Acid or Amide	0.37	0.224	0.45	0.669	0.54	0.724
2-aminobenzamide	Amino Acid or Amide	0.43	0.515	0.45	0.642	0.61	0.287
3-METHOXYTYRAMINE	Amino Acid or Amide	0.46	0.752	0.39	0.287	0.36	0.184
3-METHYLHISTAMINE	Amino Acid or Amide	0.38	0.239	0.52	0.897	0.54	0.752
3-Methylhistidine	Amino Acid or Amide	0.32	0.094	0.45	0.616	0.38	0.254
4-Acetamido-2-aminobutanoic acid	Amino Acid or Amide	0.59	0.423	0.50	0.985	0.59	0.423
4-HYDROXY-L-PROLINE	Amino Acid or Amide	0.57	0.491	0.37	0.210	0.48	0.897
4-phosphopantothenoylcysteine	Amino Acid or Amide	0.40	0.361	0.52	0.867	0.49	0.926
5-OXO-D-PROLINE	Amino Acid or Amide	0.52	0.867	0.55	0.669	0.65	0.149
Alpha-N-Phenylacetyl-L-glutamine	Amino Acid or Amide	0.49	0.956	0.42	0.445	0.48	0.867
ANILINE	Amino Acid or Amide	0.45	0.616	0.42	0.468	0.48	0.867
CITRULLINE	Amino Acid or Amide	0.36	0.184	0.62	0.270	0.53	0.780
CREATINE	Amino Acid or Amide	0.50	0.985	0.54	0.696	0.50	0.985
CREATININE	Amino Acid or Amide	0.39	0.305	0.46	0.724	0.45	0.669
D-ASPARTATE	Amino Acid or Amide	0.59	0.381	0.41	0.423	0.50	0.985
DIETHANOLAMINE	Amino Acid or Amide	0.56	0.564	0.46	0.752	0.48	0.838
DL-5-HYDROXYLYSINE	Amino Acid or Amide	0.45	0.616	0.54	0.696	0.55	0.669
D-ORNITHINE	Amino Acid or Amide	0.31	0.073	0.47	0.809	0.48	0.897
GLUTATHIONE	Amino Acid or Amide	0.46	0.752	0.61	0.305	0.51	0.956
HOMOSERINE	Amino Acid or Amide	0.55	0.616	0.62	0.254	0.67	0.110
L-ARGININE	Amino Acid or Amide	0.45	0.642	0.52	0.838	0.49	0.956
L-ASPARAGINE	Amino Acid or Amide	0.45	0.616	0.40	0.341	0.52	0.897
L-CYSTATHIONINE	Amino Acid or Amide	0.55	0.616	0.46	0.696	0.51	0.956
L-CYSTINE	Amino Acid or Amide	0.46	0.752	0.47	0.809	0.46	0.752
LEUCINE	Amino Acid or Amide	0.58	0.468	0.61	0.323	0.62	0.270
L-GLUTAMINE	Amino Acid or Amide	0.52	0.897	0.60	0.341	0.58	0.468
L-HISTIDINE	Amino Acid or Amide	0.67	0.110	0.63	0.224	0.65	0.160
L-HISTIDINOL	Amino Acid or Amide	0.34	0.119	0.45	0.616	0.38	0.254
L-ISOLEUCINE	Amino Acid or Amide	0.51	0.926	0.64	0.184	0.61	0.287
L-KYNURENINE	Amino Acid or Amide	0.32	0.086	0.46	0.752	0.49	0.956

LL-2.6-DIAMINOHEPTANEDIOATE	Amino Acid or Amide	0.65	0.160	0.48	0.897	0.68	0.086
L-LYSINE	Amino Acid or Amide	0.40	0.361	0.55	0.616	0.60	0.341
L-METHIONINE	Amino Acid or Amide	0.43	0.515	0.61	0.305	0.61	0.323
L-N.Gamma.-monomethylarginine	Amino Acid or Amide	0.57	0.515	0.63	0.239	0.63	0.224
L-PHENYLALANINE	Amino Acid or Amide	0.55	0.669	0.46	0.752	0.67	0.110
L-PIPECOLIC ACID	Amino Acid or Amide	0.61	0.305	0.57	0.539	0.59	0.381
L-PROLINE	Amino Acid or Amide	0.52	0.897	0.66	0.138	0.65	0.149
L-SERINE	Amino Acid or Amide	0.46	0.752	0.34	0.119	0.51	0.926
L-THREONINE	Amino Acid or Amide	0.51	0.956	0.46	0.724	0.56	0.590
L-TRYPTOPHAN	Amino Acid or Amide	0.56	0.590	0.45	0.616	0.67	0.102
L-TYROSINE	Amino Acid or Amide	0.52	0.897	0.55	0.616	0.61	0.305
L-VALINE	Amino Acid or Amide	0.56	0.590	0.51	0.926	0.56	0.564
N(PAI)-METHYL-L-HISTIDINE	Amino Acid or Amide	0.33	0.102	0.52	0.867	0.45	0.669
N8-ACETYLSPERMIDINE	Amino Acid or Amide	0.53	0.809	0.49	0.926	0.60	0.361
NEPSILON.NEPSILON.NEPSILON-TRIMETHYLLYSINE	Amino Acid or Amide	0.57	0.515	0.45	0.616	0.54	0.752
NG, NG-dimethyl-L-arginine	Amino Acid or Amide	0.48	0.897	0.46	0.752	0.48	0.897
NICOTINAMIDE	Amino Acid or Amide	0.49	0.956	0.68	0.086	0.54	0.724
NICOTINAMIDE MONONUCLEOTIDE	Amino Acid or Amide	0.71	0.039	0.55	0.669	0.69	0.067
nicotinate beta-D-ribonucleotide	Amino Acid or Amide	0.50	0.985	0.56	0.590	0.46	0.752
Nicotinuric acid	Amino Acid or Amide	0.38	0.239	0.45	0.616	0.43	0.539
N-METHYL-D-ASPARTIC ACID	Amino Acid or Amide	0.52	0.897	0.61	0.323	0.54	0.752
N-METHYL-L-GLUTAMATE	Amino Acid or Amide	0.55	0.616	0.51	0.956	0.55	0.616
N-Methylnicotinamide	Amino Acid or Amide	0.39	0.323	0.52	0.867	0.49	0.926
N-Undecanoylglycine	Amino Acid or Amide	0.32	0.086	0.42	0.468	0.36	0.196
O-ACETYL-L-SERINE	Amino Acid or Amide	0.38	0.270	0.36	0.184	0.34	0.119
O-PHOSPHO-L-SERINE	Amino Acid or Amide	0.49	0.956	0.25	0.014	0.49	0.956
PICOLINIC ACID	Amino Acid or Amide	0.36	0.184	0.52	0.897	0.48	0.838
Pyroglutamic acid	Amino Acid or Amide	0.66	0.119	0.62	0.254	0.67	0.110
QUINOLINE	Amino Acid or Amide	0.52	0.838	0.46	0.752	0.60	0.361
THYROXINE	Amino Acid or Amide	0.69	0.067	0.63	0.210	0.76	0.011
TYRAMINE	Amino Acid or Amide	0.45	0.616	0.51	0.926	0.51	0.926
UROCANATE	Amino Acid or Amide	0.36	0.184	0.36	0.184	0.36	0.171
6-PHOSPHOGLUCONIC ACID	Carbohydrate	0.38	0.270	0.54	0.724	0.34	0.128
ALPHA-D-GLUCOSE	Carbohydrate	0.61	0.305	0.53	0.809	0.57	0.539
D-GALACTOSE	Carbohydrate	0.73	0.026	0.57	0.539	0.72	0.035
D-GLUCOSE-6-PHOSPHATE	Carbohydrate	0.35	0.160	0.49	0.956	0.29	0.047

GALACTITOL	Carbohydrate	0.63	0.239	0.44	0.590	0.48	0.897
MALTOSE	Carbohydrate	0.48	0.867	0.41	0.381	0.51	0.956
MELIBIOSE	Carbohydrate	0.53	0.809	0.58	0.468	0.60	0.341
N-acetyllactosamine	Carbohydrate	0.41	0.423	0.39	0.323	0.50	0.985
N-ACETYLNEURAMINATE	Carbohydrate	0.36	0.184	0.39	0.323	0.39	0.287
Acetylcarnitine	Carnitine	0.53	0.780	0.47	0.809	0.43	0.515
Acylcarnitine(C10:0)	Carnitine	0.50	0.985	0.41	0.402	0.44	0.590
Acylcarnitine(C14:0)	Carnitine	0.50	0.985	0.41	0.402	0.53	0.809
Acylcarnitine(C16:0)	Carnitine	0.46	0.752	0.58	0.468	0.54	0.696
Acylcarnitine(C18:0)	Carnitine	0.48	0.838	0.53	0.809	0.48	0.897
Acylcarnitine(C18:1)	Carnitine	0.53	0.809	0.60	0.341	0.49	0.956
DEOXYCARNITINE	Carnitine	0.63	0.239	0.55	0.616	0.58	0.468
Dodecanedioylcarnitine	Carnitine	0.45	0.616	0.45	0.616	0.44	0.590
Glutarylcarnitine	Carnitine	0.47	0.809	0.47	0.780	0.44	0.590
Hexanoylcarnitine	Carnitine	0.63	0.224	0.51	0.956	0.53	0.809
Hydroxybutyrylcarnitine	Carnitine	0.39	0.287	0.44	0.564	0.34	0.119
L-carnitine	Carnitine	0.51	0.926	0.41	0.423	0.45	0.642
O-ACETYL-L-CARNITINE	Carnitine	0.57	0.491	0.47	0.809	0.48	0.897
Octanoylcarnitine	Carnitine	0.55	0.642	0.44	0.590	0.50	1.000
Propionylcarnitine	Carnitine	0.61	0.287	0.54	0.724	0.57	0.539
Tiglylcarnitine	Carnitine	0.49	0.926	0.51	0.956	0.47	0.809
Cer(41:1)	Ceramide	0.55	0.616	0.52	0.867	0.59	0.402
Aspartyl-Threonine	Dipeptide	0.55	0.642	0.43	0.515	0.52	0.867
Glycyl-threonine	Dipeptide	0.42	0.468	0.64	0.184	0.58	0.468
Histidinyl-Proline	Dipeptide	0.54	0.724	0.56	0.564	0.75	0.015
1-(Hydroxymethyl)-5,5-dimethyl-2,4-imidazolidinedione_exogenous	Exogenous	0.54	0.696	0.63	0.239	0.65	0.160
13-Nor-6-eremophilene-8,11-dione_exogenous	Exogenous	0.40	0.341	0.44	0.590	0.39	0.287
Benzofuran	Exogenous	0.52	0.867	0.56	0.590	0.62	0.254
cis-Quinceoxepane	Exogenous	0.58	0.445	0.50	0.985	0.50	0.985
Metformin	Exogenous	0.53	0.780	0.52	0.897	0.47	0.809
Trazodone	Exogenous	0.55	0.669	0.30	0.051	0.39	0.323
4,7-dioxo-octanoic acid	Fatty Acid	0.59	0.423	0.35	0.149	0.61	0.323
cis-9, cis-12-Octadecadienoic acid+	Fatty Acid	0.65	0.149	0.46	0.696	0.59	0.423
Dodecanoic acid	Fatty Acid	0.64	0.184	0.54	0.696	0.57	0.515
HEPTANOIC ACID	Fatty Acid	0.54	0.724	0.51	0.956	0.53	0.780
LINOLEATE	Fatty Acid	0.62	0.254	0.48	0.838	0.52	0.838

myristoleic acid	Fatty Acid	0.54	0.752	0.38	0.270	0.64	0.184
GlcCer(39:2)	Glycosphingolipid	0.74	0.019	0.77	0.008	0.82	0.001
LacCer(32:0)	Glycosphingolipid	0.74	0.019	0.76	0.012	0.82	0.001
LacCer(32:1)	Glycosphingolipid	0.69	0.067	0.69	0.073	0.78	0.007
LacCer(34:1)	Glycosphingolipid	0.64	0.171	0.67	0.102	0.68	0.094
NeuAc?2-3Gal?1-4Glc?-Cer(d18:1/16:0) [Trihexosylcer(d18:1/16:0]	Glycosphingolipid	0.49	0.956	0.53	0.809	0.51	0.926
NeuAc?2-3Gal?-Cer(34:1) [DihexosylCer(34:1)]	Glycosphingolipid	0.62	0.254	0.60	0.361	0.66	0.138
NeuAc?2-3Gal?-Cer(36:1) [DihexosylCer(36:1)]	Glycosphingolipid	0.62	0.270	0.60	0.341	0.66	0.119
trihexosylceramide(34:1)	Glycosphingolipid	0.64	0.171	0.55	0.642	0.55	0.642
CORTICOSTERONE	Hormone	0.65	0.149	0.48	0.897	0.64	0.196
CORTISOL	Hormone	0.54	0.724	0.39	0.305	0.74	0.021
CORTISOL 21-ACETATE	Hormone	0.54	0.724	0.37	0.224	0.57	0.539
CORTISONE	Hormone	0.55	0.669	0.48	0.867	0.64	0.184
NORADRENALINE	Hormone	0.35	0.160	0.43	0.539	0.50	0.985
Putative_Progesterone 3-biotin	Hormone	0.71	0.043	0.73	0.023	0.79	0.005
SEROTONIN	Hormone	0.52	0.838	0.47	0.809	0.66	0.138
Glycerophosphocholine	Lipid	0.57	0.491	0.58	0.445	0.59	0.402
PHOSPHOCHOLINE	Lipid	0.50	1.000	0.64	0.196	0.52	0.838
SN-GLYCEROL 3-PHOSPHATE	Lipid	0.50	0.985	0.57	0.515	0.52	0.897
LPC(14:0)	Lysophosphatidylcholine	0.58	0.468	0.50	0.985	0.59	0.423
LPC(15:0)	Lysophosphatidylcholine	0.54	0.752	0.56	0.590	0.58	0.468
LPC(16:0)	Lysophosphatidylcholine	0.55	0.616	0.51	0.926	0.61	0.323
LPC(16:1)	Lysophosphatidylcholine	0.54	0.696	0.52	0.838	0.54	0.696
LPC(17:0)	Lysophosphatidylcholine	0.61	0.287	0.48	0.867	0.62	0.254
LPC(17:1)	Lysophosphatidylcholine	0.54	0.752	0.52	0.897	0.54	0.724
LPC(18:0)	Lysophosphatidylcholine	0.60	0.341	0.55	0.642	0.63	0.224
LPC(18:2)	Lysophosphatidylcholine	0.50	1.000	0.51	0.956	0.53	0.780
LPC(18:3)	Lysophosphatidylcholine	0.47	0.780	0.44	0.564	0.52	0.897
LPC(20:1)	Lysophosphatidylcholine	0.54	0.724	0.57	0.491	0.49	0.926
LPC(20:2)	Lysophosphatidylcholine	0.56	0.564	0.57	0.539	0.52	0.838
LPC(20:3)	Lysophosphatidylcholine	0.50	1.000	0.54	0.752	0.47	0.809
LPC(20:4)	Lysophosphatidylcholine	0.44	0.590	0.52	0.838	0.42	0.468
LPC(20:5)	Lysophosphatidylcholine	0.55	0.642	0.44	0.564	0.54	0.724
LPC(22:5)	Lysophosphatidylcholine	0.57	0.515	0.58	0.445	0.53	0.809
LPC(22:6)	Lysophosphatidylcholine	0.51	0.926	0.50	1.000	0.52	0.897
LPC(26:0)	Lysophosphatidylcholine	0.61	0.323	0.61	0.287	0.74	0.019

LPC(P-16:0)	Lysophosphatidylcholine	0.52	0.838	0.63	0.224	0.55	0.669
LPC(P-18:0)	Lysophosphatidylcholine	0.68	0.086	0.59	0.381	0.62	0.254
LPC(P-18:0/0:0) or LPC(O-18:1)	Lysophosphatidylcholine	0.62	0.254	0.61	0.305	0.58	0.468
LPE(18:1)	Lysophosphatidylethanolamine	0.43	0.539	0.48	0.867	0.46	0.696
LPE(18:2)	Lysophosphatidylethanolamine	0.45	0.669	0.54	0.696	0.45	0.669
LPE(19:0)	Lysophosphatidylethanolamine	0.66	0.128	0.61	0.287	0.61	0.305
LPE(22:0)	Lysophosphatidylethanolamine	0.65	0.149	0.63	0.239	0.68	0.080
LPE(22:6)	Lysophosphatidylethanolamine	0.62	0.254	0.49	0.956	0.54	0.696
1-linoleoylglycerol	Monoacylglycerol	0.67	0.102	0.60	0.361	0.69	0.067
Monoelaidin	Monoacylglycerol	0.57	0.515	0.55	0.642	0.55	0.642
1-METHYLADENOSINE	Purine/Pyrimidine	0.53	0.780	0.44	0.590	0.54	0.752
5,6-dihydrouridine	Purine/Pyrimidine	0.43	0.491	0.44	0.590	0.41	0.402
ADENOSINE 3'.5'-DIPHOSPHATE	Purine/Pyrimidine	0.49	0.956	0.56	0.564	0.43	0.515
ADENOSINE 5'-MONOPHOSPHATE	Purine/Pyrimidine	0.52	0.897	0.57	0.539	0.50	1.000
Alloxan	Purine/Pyrimidine	0.57	0.491	0.66	0.119	0.69	0.067
GUANOSINE 3'.5'-CYCLIC MONOPHOSPHATE	Purine/Pyrimidine	0.68	0.080	0.59	0.423	0.64	0.171
HYPOXANTHINE	Purine/Pyrimidine	0.63	0.210	0.55	0.642	0.71	0.043
PARAXANTHINE	Purine/Pyrimidine	0.66	0.119	0.62	0.254	0.79	0.005
UDP-GlcNAc	Purine/Pyrimidine	0.41	0.423	0.52	0.838	0.43	0.539
URIDINE	Purine/Pyrimidine	0.71	0.043	0.60	0.361	0.68	0.086
XANTHINE	Purine/Pyrimidine	0.48	0.867	0.52	0.897	0.57	0.515
XANTHURENIC ACID	Purine/Pyrimidine	0.60	0.361	0.63	0.239	0.66	0.119
1-HYDROXY-2-NAPHTHOATE	Organic Acid	0.43	0.539	0.47	0.780	0.48	0.867
2-HYDROXY-4-(METHYLTHIO)BUTYRIC ACID	Organic Acid	0.50	1.000	0.41	0.381	0.43	0.515
3-(4-Hydroxyphenyl)propionic acid	Organic Acid	0.57	0.491	0.50	0.985	0.53	0.780
4-HYDROXY-2-QUINOLINECARBOXYLIC ACID	Organic Acid	0.73	0.026	0.57	0.515	0.62	0.270
CITRATE	Organic Acid	0.57	0.491	0.67	0.102	0.59	0.402
Coumaric Acid	Organic Acid	0.50	0.985	0.55	0.616	0.58	0.445
Hydroxyphenanthrene	Organic Acid	0.71	0.043	0.54	0.752	0.73	0.029
Indole-3-lactic acid	Organic Acid	0.56	0.590	0.54	0.696	0.51	0.926
Indoleacrylic acid	Organic Acid	0.51	0.926	0.47	0.809	0.68	0.094
Pyrrole-2-carboxylic acid	Organic Acid	0.44	0.590	0.45	0.642	0.45	0.642
URATE	Organic Acid	0.48	0.838	0.51	0.956	0.49	0.926
BETAINE	Other	0.54	0.752	0.49	0.956	0.45	0.669
BILIVERDIN	Other	0.71	0.047	0.67	0.102	0.66	0.119
CAFFEINE	Other	0.57	0.539	0.61	0.305	0.65	0.160

DETHIOBIOTIN	Other	0.57	0.491	0.53	0.780	0.66	0.128
INDOLE-3-ACETALDEHYDE	Other	0.57	0.515	0.47	0.780	0.66	0.128
Indoleacetaldehyde	Other	0.52	0.838	0.57	0.491	0.56	0.590
piperine	Other	0.45	0.669	0.51	0.926	0.51	0.956
PC(30:0)	Phosphatidylcholine	0.43	0.515	0.47	0.809	0.53	0.780
PC(31:0)	Phosphatidylcholine	0.50	0.985	0.46	0.724	0.49	0.926
PC(32:1)	Phosphatidylcholine	0.39	0.305	0.45	0.642	0.46	0.752
PC(32:2)	Phosphatidylcholine	0.56	0.590	0.68	0.094	0.61	0.323
PC(34:1)	Phosphatidylcholine	0.62	0.270	0.35	0.149	0.48	0.867
PC(34:2)	Phosphatidylcholine	0.70	0.061	0.43	0.539	0.54	0.724
PC(35:1)	Phosphatidylcholine	0.66	0.119	0.45	0.669	0.61	0.287
PC(36:2)	Phosphatidylcholine	0.73	0.026	0.47	0.780	0.73	0.023
PC(36:3)	Phosphatidylcholine	0.56	0.564	0.52	0.838	0.65	0.149
PC(36:4)	Phosphatidylcholine	0.57	0.491	0.38	0.270	0.57	0.539
PC(36:5)	Phosphatidylcholine	0.55	0.642	0.42	0.468	0.56	0.564
PC(38:5)	Phosphatidylcholine	0.55	0.669	0.46	0.724	0.55	0.616
PC(38:6)	Phosphatidylcholine	0.54	0.752	0.58	0.445	0.62	0.254
PC(38:7)	Phosphatidylcholine	0.54	0.724	0.49	0.926	0.56	0.590
PC(40:7)	Phosphatidylcholine	0.61	0.323	0.60	0.341	0.61	0.287
PC(o-30:1) or PC(p-30:0)	Phosphatidylcholine	0.56	0.564	0.46	0.724	0.59	0.381
PC(o-32:1) or PC(p-32:0)	Phosphatidylcholine	0.41	0.423	0.53	0.809	0.53	0.809
PC(o-34:4) or PC(p-34:3)	Phosphatidylcholine	0.50	0.985	0.47	0.809	0.52	0.867
PC(o-38:4) or PC(p-38:3)	Phosphatidylcholine	0.54	0.724	0.57	0.515	0.63	0.239
PC(o-38:6) or PC(p-38:6)	Phosphatidylcholine	0.56	0.590	0.59	0.423	0.55	0.616
PC(o-40:7) or PC(p-40:6)	Phosphatidylcholine	0.56	0.590	0.51	0.956	0.60	0.361
PE(36:1)	Phosphatidylethanolamine	0.65	0.149	0.58	0.445	0.70	0.056
PE(36:2)	Phosphatidylethanolamine	0.57	0.539	0.64	0.196	0.68	0.086
PE(36:4)	Phosphatidylethanolamine	0.44	0.590	0.45	0.669	0.48	0.867
PE(37:4)	Phosphatidylethanolamine	0.65	0.149	0.45	0.669	0.56	0.590
PE(38:3)	Phosphatidylethanolamine	0.69	0.073	0.60	0.361	0.73	0.023
PE(38:3)	Phosphatidylethanolamine	0.58	0.445	0.58	0.445	0.66	0.119
PE(40:6)	Phosphatidylethanolamine	0.56	0.590	0.41	0.423	0.52	0.867
PE(o-36:5) or PE(p-36:4)	Phosphatidylethanolamine	0.64	0.171	0.64	0.184	0.70	0.051
PE(p-18:0_20:4)	Phosphatidylethanolamine	0.60	0.361	0.65	0.160	0.71	0.039
PI(38:4)	Phosphatidylinositol	0.66	0.119	0.67	0.110	0.74	0.021
Putative_PI(36:2)	Phosphatidylinositol	0.72	0.032	0.76	0.012	0.76	0.012

Putative_PI(40:3)	Phosphatidylinositol	0.65	0.149	0.70	0.056	0.78	0.006
LEUKOTRIENE B4	Prostanoid	0.46	0.696	0.46	0.752	0.39	0.305
Prostaglandin A1	Prostanoid	0.75	0.014	0.55	0.642	0.64	0.184
Prostaglandin E2	Prostanoid	0.55	0.616	0.36	0.184	0.52	0.867
SM(32:1)	Sphingomyelin	0.63	0.224	0.57	0.539	0.62	0.270
SM(32:2)	Sphingomyelin	0.65	0.149	0.64	0.196	0.63	0.239
SM(34:1)	Sphingomyelin	0.50	0.985	0.59	0.423	0.55	0.616
SM(34:2)	Sphingomyelin	0.56	0.590	0.61	0.287	0.58	0.468
SM(36:1)	Sphingomyelin	0.63	0.239	0.61	0.323	0.65	0.149
SM(36:2)	Sphingomyelin	0.48	0.867	0.53	0.780	0.55	0.642
SM(40:2)	Sphingomyelin	0.73	0.029	0.65	0.160	0.72	0.032
SM(42:1)	Sphingomyelin	0.52	0.838	0.52	0.867	0.53	0.809
SM(42:3)	Sphingomyelin	0.74	0.019	0.64	0.171	0.71	0.039
7-oxo-cholesterol	Sterol	0.61	0.305	0.48	0.897	0.50	0.985
8-Deoxy-11,13-dihydroxygrosheimin	Sterol	0.44	0.564	0.53	0.809	0.35	0.149
CHOLATE	Sterol	0.66	0.119	0.51	0.926	0.58	0.445
DEOXYCHOLATE	Sterol	0.55	0.642	0.53	0.780	0.54	0.724
GLYCOCHOLATE	Sterol	0.55	0.616	0.50	0.985	0.52	0.838
sterol	Sterol	0.62	0.254	0.55	0.669	0.60	0.361
TG(16:0_18:1_18:2)	Triacylglycerol	0.50	0.985	0.51	0.956	0.47	0.809
TG(44:0)	Triacylglycerol	0.58	0.468	0.47	0.809	0.54	0.696
TG(45:0)	Triacylglycerol	0.61	0.323	0.47	0.780	0.59	0.423
TG(46:0)	Triacylglycerol	0.62	0.254	0.48	0.897	0.64	0.184
TG(46:1)	Triacylglycerol	0.58	0.468	0.53	0.809	0.55	0.669
TG(48:1)	Triacylglycerol	0.55	0.642	0.49	0.926	0.56	0.564
TG(48:2)	Triacylglycerol	0.57	0.539	0.53	0.780	0.55	0.642
TG(49:0)	Triacylglycerol	0.59	0.381	0.44	0.590	0.55	0.616
TG(49:1)	Triacylglycerol	0.54	0.696	0.41	0.381	0.55	0.616
TG(50:2)	Triacylglycerol	0.47	0.780	0.51	0.926	0.50	1.000
TG(50:3)	Triacylglycerol	0.55	0.669	0.54	0.752	0.53	0.809
TG(51:3)	Triacylglycerol	0.56	0.590	0.55	0.669	0.54	0.696
TG(51:4)	Triacylglycerol	0.57	0.515	0.54	0.724	0.57	0.491
TG(52:0)	Triacylglycerol	0.54	0.752	0.50	1.000	0.53	0.780
TG(52:1)	Triacylglycerol	0.46	0.724	0.46	0.752	0.50	1.000
TG(52:4)	Triacylglycerol	0.50	0.985	0.54	0.752	0.52	0.897
TG(52:7)	Triacylglycerol	0.61	0.323	0.47	0.780	0.59	0.423

TG(53:1)	Triacylglycerol	0.57	0.515	0.43	0.539	0.59	0.423
TG(53:2)	Triacylglycerol	0.54	0.724	0.46	0.696	0.51	0.926
TG(54:1)	Triacylglycerol	0.59	0.381	0.51	0.956	0.54	0.724
TG(54:2)	Triacylglycerol	0.43	0.515	0.52	0.897	0.44	0.564
TG(54:3)	Triacylglycerol	0.40	0.361	0.49	0.956	0.46	0.724
TG(54:5)	Triacylglycerol	0.43	0.539	0.52	0.838	0.50	0.985
TG(54:6)	Triacylglycerol	0.47	0.809	0.48	0.897	0.52	0.897
TG(55:2)	Triacylglycerol	0.48	0.867	0.43	0.539	0.52	0.897
TG(56:7)	Triacylglycerol	0.54	0.752	0.52	0.867	0.57	0.515
TG(57:1)	Triacylglycerol	0.44	0.564	0.48	0.838	0.50	1.000
TG(58:9)	Triacylglycerol	0.54	0.724	0.50	0.985	0.59	0.423
TG(61:13)	Triacylglycerol	0.55	0.642	0.32	0.086	0.54	0.752
TG(65:11)	Triacylglycerol	0.43	0.515	0.44	0.590	0.57	0.491
TG(65:11)_iso	Triacylglycerol	0.52	0.838	0.45	0.669	0.59	0.402
3-cis-Hydroxy-b,e-Caroten-3'-one	Vitamin	0.68	0.080	0.49	0.956	0.66	0.128
4-PYRIDOXATE	Vitamin	0.35	0.149	0.48	0.838	0.49	0.926
Choline	Vitamin	0.51	0.956	0.46	0.724	0.49	0.956
D-PANTOTHENIC ACID	Vitamin	0.38	0.270	0.43	0.515	0.50	0.985
FOLIC ACID	Vitamin	0.53	0.780	0.42	0.445	0.49	0.956
RIBOFLAVIN	Vitamin	0.62	0.254	0.54	0.724	0.55	0.669

[1] AUC (Baseline Aggressive vs Baseline Indolent);
[2] AUC (12 Month Aggressive vs 12 Month Indolent)
[3] AUC (12 Month Aggressive vs Baseline Indolent)
[#] Statistical significance (2-sided) was determined by Wilcoxon Rank-Sum Test; *p*-values were not adjusted for multiple-comparison testing

Supplementary Table 2. Hazard ratios of individual metabolites for progression-free survival in the MDACC validation cohort.

MDACC Validation Cohort

Metabolite	Domain	HR#	2-sided *p*-value‡	1-sided *p*-value	Lower 95 CI	Upper 95 CI
Acetylcarnitine	Acylcarnitine	1.4	0.123	0.062	0.92	2.09
Acylcarnitine(C14:0)	Acylcarnitine	1.1	0.616	0.308	0.72	1.73
Acylcarnitine(C16:0)	Acylcarnitine	1.1	0.693	0.346	0.61	2.09
Acylcarnitine(C18:0)	Acylcarnitine	1.3	0.510	0.255	0.64	2.44
Acylcarnitine(C18:1)	Acylcarnitine	1.2	0.499	0.249	0.70	2.11
Acylcarnitine(C18:2n-6)	Acylcarnitine	1.1	0.639	0.320	0.69	1.85
Acylcarnitine(C8:0)	Acylcarnitine	1.0	0.696	0.348	0.93	1.12
Dodecanedioylcarnitine	Acylcarnitine	1.2	0.506	0.253	0.72	1.97
LAUROYLCARNITINE	Acylcarnitine	1.1	0.298	0.149	0.89	1.46
O-butanoyl-R-carnitine	Acylcarnitine	1.2	0.599	0.299	0.68	1.95
O-decanoylcarnitine	Acylcarnitine	1.0	0.487	0.243	0.93	1.17
O-hexanoyl-R-carnitine	Acylcarnitine	1.2	0.412	0.206	0.82	1.63
O-octanoyl-R-carnitine	Acylcarnitine	1.0	0.620	0.310	0.91	1.17
Ceramide(18:1_24:1)	Ceramide	0.8	0.474	0.237	0.43	1.48
Ceramide(18:2_16:0)	Ceramide	1.3	0.550	0.275	0.55	3.07
Ceramide(30:1)	Ceramide	1.7	0.396	0.198	0.52	5.21
Ceramide(32:1)	Ceramide	0.9	0.745	0.373	0.34	2.16
Ceramide(34:1)	Ceramide	1.8	0.286	0.143	0.62	5.18
Ceramide(39:1)	Ceramide	0.9	0.819	0.410	0.52	1.68
Ceramide(40:0)	Ceramide	0.9	0.628	0.314	0.56	1.43
Ceramide(40:1)	Ceramide	1.0	0.880	0.440	0.61	1.77
Ceramide(40:2)	Ceramide	0.9	0.622	0.311	0.51	1.50
Ceramide(41:1)	Ceramide	1.1	0.850	0.425	0.56	2.02
Ceramide(42:0)	Ceramide	0.9	0.592	0.296	0.54	1.42
Ceramide(42:1)	Ceramide	1.2	0.523	0.262	0.68	2.14
Ceramide(42:2)	Ceramide	0.8	0.526	0.263	0.33	1.77
Ceramide(43:1)	Ceramide	0.9	0.827	0.414	0.46	1.86
Cholesterol Ester(16:1)	Cholesterol Ester	1.0	0.867	0.434	0.67	1.41
Cholesterol Ester(20:4)	Cholesterol Ester	2.6	0.004	0.002	1.36	4.83
Cholesterol Ester(20:5)	Cholesterol Ester	1.1	0.406	0.203	0.85	1.51
Cholesterol Ester(22:6)	Cholesterol Ester	1.1	0.805	0.403	0.72	1.54
Diacylglycerol(32:0)	Diacylglycerol	0.9	0.298	0.149	0.74	1.10

Diacylglycerol(34:0)	Diacylglycerol	0.9	0.258	0.129	0.67	1.12
Diacylglycerol(34:1)	Diacylglycerol	0.9	0.384	0.192	0.66	1.18
Diacylglycerol(34:2)	Diacylglycerol	0.9	0.524	0.262	0.67	1.22
Diacylglycerol(36:3)	Diacylglycerol	0.9	0.420	0.210	0.58	1.26
Diacylglycerol(36:4)	Diacylglycerol	1.0	0.981	0.490	0.74	1.36
Diacylglycerol(37:7)	Diacylglycerol	0.9	0.333	0.166	0.76	1.10
LEUKOTRIENE B4	Eicosanoid	0.3	0.166	0.083	0.07	1.57
Prostaglandin D2;Prostaglandin E2	Eicosanoid	4.4	0.047	0.024	1.02	18.99
Prostaglandin D2;Prostaglandin E2	Eicosanoid	1.5	0.463	0.232	0.52	4.23
4,7-dioxo-octanoic acid	Free Fatty Acid	0.8	0.564	0.282	0.31	1.89
Free Fatty Acid (18:1)	Free Fatty Acid	1.1	0.429	0.214	0.92	1.23
Free Fatty Acid (18:2) (linoleic acid)	Free Fatty Acid	1.1	0.162	0.081	0.96	1.32
Free Fatty Acid (20:4) (arachidonic acid)	Free Fatty Acid	1.2	0.145	0.073	0.93	1.64
Free Fatty Acid (22:6) (docosahexaenoic acid)	Free Fatty Acid	1.0	0.953	0.477	0.80	1.27
Glucosyl/GalactosylCer(40:0)	Glycosphingolipid	3.3	0.030	0.015	1.12	9.40
GlucosylCeramide(42:1)	Glycosphingolipid	1.3	0.501	0.251	0.63	2.57
LactosylCeramide(18:1/16:0)	Glycosphingolipid	1.6	0.247	0.123	0.72	3.53
Lactosylceramide(18:1/20:4)	Glycosphingolipid	1.0	0.909	0.455	0.46	2.01
LactosylCeramide(32:0)	Glycosphingolipid	2.0	0.016	0.008	1.14	3.51
Lactosylceramide(32:0)	Glycosphingolipid	1.4	0.388	0.194	0.68	2.75
LactosylCeramide(34:1)	Glycosphingolipid	1.8	0.199	0.100	0.75	4.09
LactosylCeramide(36:0)	Glycosphingolipid	1.9	0.039	0.019	1.03	3.52
LactosylCeramide(36:0)	Glycosphingolipid	2.9	0.055	0.027	0.98	8.36
Trihexosylceramide(34:1)	Glycosphingolipid	2.6	0.047	0.023	1.01	6.54
Trihexosylceramide(40:1)	Glycosphingolipid	2.2	0.069	0.034	0.94	5.16
Lysophosphatidylcholine(14:0)	Lysophospholipid	0.9	0.727	0.363	0.55	1.52
Lysophosphatidylcholine(15:0)	Lysophospholipid	1.1	0.887	0.444	0.46	2.44
Lysophosphatidylcholine(15:1)	Lysophospholipid	1.0	0.979	0.489	0.56	1.77
Lysophosphatidylcholine(16:0)	Lysophospholipid	1.0	0.948	0.474	0.45	2.34
Lysophosphatidylcholine(16:1)	Lysophospholipid	1.0	0.919	0.460	0.59	1.81
Lysophosphatidylcholine(17:0)	Lysophospholipid	1.3	0.373	0.187	0.71	2.46
Lysophosphatidylcholine(17:1)	Lysophospholipid	1.3	0.390	0.195	0.70	2.54
Lysophosphatidylcholine(17:2)	Lysophospholipid	1.4	0.219	0.109	0.81	2.53
Lysophosphatidylcholine(18:0)	Lysophospholipid	1.3	0.475	0.237	0.63	2.69
Lysophosphatidylcholine(18:1)	Lysophospholipid	1.5	0.201	0.100	0.82	2.60
Lysophosphatidylcholine(18:2)	Lysophospholipid	1.4	0.225	0.113	0.81	2.45
Lysophosphatidylcholine(18:3)	Lysophospholipid	1.4	0.125	0.062	0.92	2.05

Lysophosphatidylcholine(20:0)	Lysophospholipid	1.2	0.258	0.129	0.86	1.75
Lysophosphatidylcholine(20:1)	Lysophospholipid	1.2	0.535	0.268	0.68	2.11
Lysophosphatidylcholine(20:2)	Lysophospholipid	1.4	0.420	0.210	0.63	3.09
Lysophosphatidylcholine(20:3)	Lysophospholipid	1.1	0.728	0.364	0.69	1.69
Lysophosphatidylcholine(20:4)	Lysophospholipid	1.1	0.402	0.201	0.84	1.57
Lysophosphatidylcholine(20:5)	Lysophospholipid	1.1	0.597	0.299	0.85	1.34
Lysophosphatidylcholine(22:4)	Lysophospholipid	1.2	0.456	0.228	0.76	1.85
Lysophosphatidylcholine(22:5)	Lysophospholipid	1.1	0.489	0.244	0.80	1.59
Lysophosphatidylcholine(22:6)	Lysophospholipid	1.2	0.357	0.179	0.83	1.66
Lysophosphatidylcholine(24:0)	Lysophospholipid	1.4	0.365	0.183	0.67	3.00
Lysophosphatidylcholine(26:0)	Lysophospholipid	1.8	0.280	0.140	0.62	5.29
Lysophosphatidylethanolamine(16:0)	Lysophospholipid	1.2	0.543	0.271	0.62	2.49
Lysophosphatidylethanolamine(18:0)	Lysophospholipid	1.4	0.395	0.197	0.67	2.76
Lysophosphatidylethanolamine(18:1)	Lysophospholipid	1.4	0.102	0.051	0.94	2.01
Lysophosphatidylethanolamine(18:2)	Lysophospholipid	1.3	0.330	0.165	0.78	2.11
Lysophosphatidylethanolamine(20:3)	Lysophospholipid	1.9	0.124	0.062	0.84	4.27
Lysophosphatidylethanolamine(20:4)	Lysophospholipid	1.3	0.374	0.187	0.74	2.22
Lysophosphatidylethanolamine(22:0)	Lysophospholipid	1.6	0.336	0.168	0.62	4.14
Lysophosphatidylethanolamine(22:6)	Lysophospholipid	1.1	0.634	0.317	0.68	1.88
Plas_Lysophosphatidylcholine(P-18:0/0:0) or Plas_Lysophosphatidylcholine(O-18:1)	Lysophospholipid	1.1	0.822	0.411	0.57	2.04
PlasLysophosphatidylcholine(P-16:0)	Lysophospholipid	1.7	0.087	0.043	0.93	3.07
PlasLysophosphatidylethanolamine(p-22:0)	Lysophospholipid	1.5	0.268	0.134	0.75	2.84
1-linoleoylglycerol	Monoacylglycerol	0.5	0.641	0.320	0.03	8.13
2-Arachidonylglycerol	Monoacylglycerol	1.3	0.112	0.056	0.95	1.69
Monoacylglycerol(20:0/0:0/0:0)	Monoacylglycerol	1.2	0.676	0.338	0.51	2.83
Phosphatidylcholine(30:1)	Phospholipid	1.1	0.666	0.333	0.75	1.58
Phosphatidylcholine(32:0)	Phospholipid	1.2	0.559	0.279	0.60	2.58
Phosphatidylcholine(32:1)	Phospholipid	0.9	0.450	0.225	0.60	1.25
Phosphatidylcholine(32:2)	Phospholipid	0.8	0.494	0.247	0.52	1.37
Phosphatidylcholine(33:5)	Phospholipid	0.9	0.815	0.407	0.53	1.66
Phosphatidylcholine(34:1)	Phospholipid	0.9	0.799	0.399	0.33	2.33
Phosphatidylcholine(34:4)	Phospholipid	0.9	0.420	0.210	0.59	1.25
Phosphatidylcholine(35:1);Phosphatidylethanolamine(38:1)	Phospholipid	1.2	0.650	0.325	0.56	2.53
Phosphatidylcholine(35:2)	Phospholipid	1.0	0.965	0.482	0.47	2.19
Phosphatidylcholine(36:1)	Phospholipid	1.3	0.492	0.246	0.63	2.64
Phosphatidylcholine(36:2)	Phospholipid	1.2	0.818	0.409	0.34	3.93
Phosphatidylcholine(36:4)	Phospholipid	1.6	0.279	0.140	0.68	3.79

Phosphatidylcholine(36:5)	Phospholipid	1.1	0.333	0.166	0.88	1.45
Phosphatidylcholine(38:5)	Phospholipid	2.4	0.022	0.011	1.13	4.96
Phosphatidylcholine(38:6)	Phospholipid	1.0	0.906	0.453	0.55	1.97
Phosphatidylcholine(38:7)	Phospholipid	1.1	0.769	0.384	0.62	1.90
Phosphatidylcholine(40:5)	Phospholipid	1.3	0.237	0.118	0.85	1.98
Phosphatidylcholine(40:7)	Phospholipid	1.3	0.397	0.199	0.74	2.12
Phosphatidylcholine(40:8)	Phospholipid	1.4	0.352	0.176	0.70	2.73
Phosphatidylethanolamine(36:1)	Phospholipid	1.2	0.476	0.238	0.72	2.02
Phosphatidylethanolamine(36:1)	Phospholipid	1.0	0.998	0.499	0.61	1.65
Phosphatidylethanolamine(38:4)	Phospholipid	1.1	0.589	0.295	0.73	1.75
Phosphatidylglycerol(43:0)	Phospholipid	1.6	0.294	0.147	0.68	3.64
Phosphatidylserine(41:1)	Phospholipid	1.3	0.562	0.281	0.57	2.84
Plas_Phosphatidylcholine(o-30:1) or Plas_Phosphatidylcholine(p-30:0)	Phospholipid	0.8	0.615	0.307	0.28	2.13
Plas_Phosphatidylcholine(o-32:1) or Plas_Phosphatidylcholine(p-32:0)	Phospholipid	1.2	0.628	0.314	0.57	2.56
Plas_Phosphatidylcholine(o-34:1) or Plas_Phosphatidylcholine(p-34:0)	Phospholipid	1.2	0.620	0.310	0.53	2.92
Plas_Phosphatidylcholine(o-34:3) or Plas_Phosphatidylcholine(p-34:2)	Phospholipid	1.1	0.220	0.110	0.94	1.32
Plas_Phosphatidylcholine(o-38:6) or Plas_Phosphatidylcholine(p-38:5)	Phospholipid	1.9	0.084	0.042	0.92	4.03
Plas_Phosphatidylcholine(o-38:7) or Plas_Phosphatidylcholine(p-38:6)	Phospholipid	1.7	0.066	0.033	0.97	3.01
Plas_Phosphatidylcholine(o-40:6) or Plas_Phosphatidylcholine(p-40:5)	Phospholipid	1.5	0.230	0.115	0.78	2.88
Plas_Phosphatidylcholine(o-40:7) or Plas_Phosphatidylcholine(p-40:6)	Phospholipid	1.9	0.026	0.013	1.08	3.29
Plas_Phosphatidylethanolamine(o-38:5) or Plas_Phosphatidylethanolamine(p-38:4)	Phospholipid	1.4	0.198	0.099	0.85	2.24
Plas_Phosphatidylethanolamine(o-40:5) or Plas_Phosphatidylethanolamine(p-40:4)	Phospholipid	1.9	0.023	0.011	1.09	3.37
Plas_Phosphatidylethanolamine(o-40:5) or Plas_Phosphatidylethanolamine(p-40:4)	Phospholipid	1.2	0.432	0.216	0.74	2.03
Plas_Phosphatidylethanolamine(o-40:6) or Plas_Phosphatidylethanolamine(p-40:5)	Phospholipid	1.5	0.179	0.089	0.84	2.49
Plas_Phosphatidylserine(p-39:1)	Phospholipid	1.2	0.680	0.340	0.45	3.38
Plas_Phosphatidylserine(p-40:1)	Phospholipid	1.0	0.916	0.458	0.53	2.02
PlasPhosphatidylcholine(o-32:1) or PlasPhosphatidylcholine(p-32:0)	Phospholipid	2.1	0.059	0.029	0.97	4.51
PlasPhosphatidylcholine(o-34:1) or PlasPhosphatidylcholine(p-34:0)	Phospholipid	1.4	0.420	0.210	0.61	3.25
PlasPhosphatidylcholine(o-40:2) or PlasPhosphatidylcholine(p-40:1)	Phospholipid	2.4	0.036	0.018	1.06	5.56
PlasPhosphatidylcholine(o-40:7) or PlasPhosphatidylcholine(p-40:6)	Phospholipid	1.4	0.348	0.174	0.72	2.59
PlasPhosphatidylcholine(o-42:5) or PlasPhosphatidylcholine(p-42:4)	Phospholipid	1.3	0.554	0.277	0.60	2.58
PlasPhosphatidylethanolamine(o-38:5) or PlasPhosphatidylethanolamine(p-38:4)	Phospholipid	1.5	0.152	0.076	0.87	2.44
Sphingomyelin(32:1)	Sphingolipid	1.2	0.534	0.267	0.70	2.01
Sphingomyelin(32:2)	Sphingolipid	1.2	0.503	0.252	0.69	2.15
Sphingomyelin(33:1)	Sphingolipid	2.4	0.091	0.046	0.87	6.34
Sphingomyelin(33:2)	Sphingolipid	1.4	0.447	0.224	0.57	3.59
Sphingomyelin(34:0)	Sphingolipid	1.7	0.167	0.084	0.79	3.78

Sphingomyelin(34:1)	Sphingolipid	2.8	0.091	0.045	0.85	8.90
Sphingomyelin(34:2)	Sphingolipid	1.5	0.337	0.168	0.67	3.29
Sphingomyelin(36:1)	Sphingolipid	1.5	0.277	0.138	0.73	2.95
Sphingomyelin(36:2)	Sphingolipid	1.5	0.233	0.117	0.76	3.11
Sphingomyelin(36:3)	Sphingolipid	1.2	0.441	0.221	0.72	2.14
Sphingomyelin(38:1)	Sphingolipid	1.5	0.274	0.137	0.74	2.89
Sphingomyelin(39:2)	Sphingolipid	1.9	0.161	0.081	0.77	4.71
Sphingomyelin(40:1)	Sphingolipid	1.7	0.119	0.060	0.88	3.09
Sphingomyelin(40:2)	Sphingolipid	2.2	0.070	0.035	0.94	5.19
Sphingomyelin(40:3)	Sphingolipid	1.4	0.285	0.143	0.76	2.52
Sphingomyelin(41:1)	Sphingolipid	2.0	0.087	0.044	0.90	4.42
Sphingomyelin(42:1)	Sphingolipid	1.8	0.089	0.045	0.91	3.51
Sphingomyelin(42:2)	Sphingolipid	1.7	0.140	0.070	0.83	3.61
Sphingomyelin(42:3)	Sphingolipid	1.6	0.242	0.121	0.72	3.62
Sphingomyelin(43:2)	Sphingolipid	1.4	0.350	0.175	0.70	2.71
Sphingomyelin(44:2)	Sphingolipid	3.9	0.007	0.004	1.45	10.60
Triacylglycerol(16:0_18:1_18:2)	Triacylglycerol	0.4	0.459	0.229	0.05	4.04
Triacylglycerol(40:0)	Triacylglycerol	1.0	0.115	0.057	0.99	1.06
Triacylglycerol(42:2)	Triacylglycerol	1.1	0.123	0.062	0.99	1.11
Triacylglycerol(44:0)	Triacylglycerol	1.0	0.450	0.225	0.94	1.16
Triacylglycerol(44:1)	Triacylglycerol	1.0	0.795	0.397	0.87	1.12
Triacylglycerol(44:2)	Triacylglycerol	1.0	0.967	0.483	0.87	1.16
Triacylglycerol(45:0)	Triacylglycerol	1.0	0.992	0.496	0.71	1.41
Triacylglycerol(46:0)	Triacylglycerol	1.3	0.107	0.053	0.94	1.87
Triacylglycerol(46:1)	Triacylglycerol	1.0	0.636	0.318	0.85	1.10
Triacylglycerol(46:2)	Triacylglycerol	0.9	0.503	0.252	0.74	1.16
Triacylglycerol(46:3)	Triacylglycerol	1.0	0.935	0.468	0.80	1.23
Triacylglycerol(47:0)	Triacylglycerol	1.2	0.567	0.284	0.59	2.63
Triacylglycerol(47:3)	Triacylglycerol	1.0	0.981	0.490	0.72	1.40
Triacylglycerol(48:0)	Triacylglycerol	1.1	0.787	0.393	0.67	1.71
Triacylglycerol(48:1)	Triacylglycerol	1.0	0.949	0.474	0.70	1.41
Triacylglycerol(48:2)	Triacylglycerol	1.0	0.934	0.467	0.63	1.53
Triacylglycerol(48:3)	Triacylglycerol	0.9	0.660	0.330	0.66	1.30
Triacylglycerol(48:3)	Triacylglycerol	1.0	0.849	0.425	0.83	1.26
Triacylglycerol(48:4)	Triacylglycerol	1.0	0.792	0.396	0.82	1.31
Triacylglycerol(49:1)	Triacylglycerol	0.9	0.601	0.300	0.71	1.22
Triacylglycerol(49:2)	Triacylglycerol	0.8	0.275	0.138	0.62	1.15

Triacylglycerol(49:3)	Triacylglycerol	0.9	0.614	0.307	0.57	1.39
Triacylglycerol(50:1)	Triacylglycerol	1.1	0.706	0.353	0.59	2.19
Triacylglycerol(50:3)	Triacylglycerol	0.8	0.461	0.231	0.38	1.56
Triacylglycerol(50:5)	Triacylglycerol	1.1	0.617	0.308	0.74	1.67
Triacylglycerol(51:1)	Triacylglycerol	1.1	0.570	0.285	0.78	1.57
Triacylglycerol(51:2)	Triacylglycerol	0.9	0.648	0.324	0.54	1.48
Triacylglycerol(51:3)	Triacylglycerol	0.8	0.360	0.180	0.43	1.36
Triacylglycerol(51:4)	Triacylglycerol	0.9	0.685	0.342	0.63	1.36
Triacylglycerol(52:2)	Triacylglycerol	1.0	0.943	0.471	0.22	4.06
Triacylglycerol(52:4)	Triacylglycerol	0.7	0.575	0.288	0.16	2.79
Triacylglycerol(52:7)	Triacylglycerol	1.5	0.092	0.046	0.94	2.34
Triacylglycerol(53:1)	Triacylglycerol	0.9	0.624	0.312	0.66	1.29
Triacylglycerol(53:2)	Triacylglycerol	1.1	0.657	0.328	0.72	1.70
Triacylglycerol(53:3)	Triacylglycerol	0.8	0.580	0.290	0.44	1.59
Triacylglycerol(53:4)	Triacylglycerol	0.9	0.755	0.378	0.56	1.53
Triacylglycerol(54:3)	Triacylglycerol	1.0	0.879	0.440	0.60	1.56
Triacylglycerol(54:4)	Triacylglycerol	1.7	0.320	0.160	0.61	4.54
Triacylglycerol(54:6)	Triacylglycerol	1.3	0.322	0.161	0.77	2.18
Triacylglycerol(55:1)	Triacylglycerol	1.2	0.602	0.301	0.66	2.06
Triacylglycerol(55:2)	Triacylglycerol	0.5	0.246	0.123	0.19	1.52
Triacylglycerol(55:2)	Triacylglycerol	1.0	0.887	0.444	0.52	1.77
Triacylglycerol(56:7)	Triacylglycerol	1.1	0.732	0.366	0.80	1.37
Triacylglycerol(56:9)	Triacylglycerol	1.4	0.032	0.016	1.03	1.97
Triacylglycerol(57:3)	Triacylglycerol	1.2	0.736	0.368	0.36	4.33
Triacylglycerol(57:8)	Triacylglycerol	1.1	0.652	0.326	0.75	1.58
Triacylglycerol(58:9)	Triacylglycerol	1.3	0.063	0.031	0.99	1.68
Triacylglycerol(59:4)	Triacylglycerol	1.3	0.419	0.210	0.67	2.60
Triacylglycerol(59:5)	Triacylglycerol	1.3	0.330	0.165	0.77	2.19
Triacylglycerol(60:3)	Triacylglycerol	1.1	0.892	0.446	0.46	2.46
Triacylglycerol(61:3)	Triacylglycerol	1.0	0.955	0.477	0.45	2.32
Triacylglycerol(61:6)	Triacylglycerol	1.4	0.347	0.174	0.68	2.98
Triacylglycerol(61:7)	Triacylglycerol	1.4	0.239	0.119	0.79	2.59
Triacylglycerol(65:11)	Triacylglycerol	1.2	0.177	0.088	0.91	1.64

treated as continuous variables; ‡ p-values
Abbrev. CI: Confidence Interval

Supplementary Table 3. Multivariate Cox-proportional hazard models for the Cav-1-sphingolipid signature and its association with disease progression free-survival. Cox proportional hazard models using a plasma Cav-1-sphingolipid signature cut-off value of 4.33. Optimal cut-off values for plasma Cav-1-sphingolipid signature were derived using log rank statistic based methods as described by Contal and O'Quigley[4]. Age, 5-α reductase treatment and baseline tumor volume (Risk Group 1: 1 positive biopsy core with tumor focus of <3.0mm in Gleason 3+3=6 patients or <2.0mm in Gleason 3+4=7 patients; Risk Group 2: >1 core or baseline tumor length greater than those of Risk Group 1) were included as co-variables based on a backward stepwise selection method (likelihood ratio).

		**Univariable**			**Multivariable†**	
**Variable**	**HR**	**95% CI**	**2-sided P**	**HR**	**95% CI**	**2-sided P**
Age						
< 64		Reference			Reference	
≥ 64	1.66	1.11-2.48	0.014	1.68	1.12-2.53	0.013
BMI^	1.02	0.40-2.64	0.965		-	
PSA Density‡	3.77	0.99-14.44	0.053		-	
5-ARI treatment						
No		Reference			Reference	
Yes	0.50	0.23-1.07	0.074	0.37	0.17-0.82	0.014
Risk Group						
I		Reference			Reference	
II	2.94	1.75-4.96	<0.001	2.60	1.54-4.38	<0.001
Sphingolipid Signature						
Below Cutoff (≤ 4.33)		Reference			Reference	
Above Cutoff (> 4.33)	2.62	1.71-4.01	<0.001	2.70	1.75-4.16	<0.001

† Variables included into the equation after selection using a backward stepwise method (likelihood ratio)
^ per unit log 2 increase
‡ per unit increase

Supplementary Table 4. Patient characteristics for MDACC discovery cohort.

	Total	Cases	Controls	*P*
N	32	16	16	
Age, y	64.4 ± 7.9	64.3 ± 8.2	64.4 ± 7.9	0.9548
BMI, kg/m^2	27.0 ± 3.9	27.3 ± 3.5	26.7 ± 4.3	0.7219
Family history of PC				
1st degree	8 (25)	3 (18.8)	5 (31.3)	0.6851
2nd degree	4 (12.5)	3 (18.8)	1 (6.3)	0.5996
Hypertension	13 (40.6)	5 (31.3)	8 (50)	0.4725
Diabetes	5 (15.6)	3 (18.8)	2 (12.5)	1
Smoking (ever smoker)	14 (43.8)	8 (50)	6 (37.5)	0.4757
5ARI	3 (9.4)	0 (0)	3 (18.8)	0.2258
Statins	10 (31.3)	4 (25)	6 (37.5)	0.7043
PSA	3.8 ± 2.1	3.7 ± 1.8	3.9 ± 2.3	0.9849
Testosterone	383.7 ± 179.2	400.9 ± 197.0	366.5 ± 164.1	0.6922
TRUS (Total)	42.0 ± 18.1	41.8 ± 19.6	42.2 ± 17.3	0.771
TRUS (TZ)	18.7 ± 11.0	19.2 ± 11.3	18.3 ± 11.0	0.8264
Summation Total Tumor Length (mm)	4.6 ± 4.9	4.8 ± 3.9	4.5 ± 5.9	0.4249
Highest Gleason Score				1
3+3	24 (75)	12 (75)	12 (75)	
3+4	7 (21.9)	3 (18.8)	4 (25)	
4+3	1 (3.1)	1 (6.3)	0 (0)	

For continuous variables, *P*-values were determined by 2-sided Wilcoxon rank sum test; for categorical variables, *P*-values were determined by 2-sided Fisher's Exact Test (binary variables) or Chi-square test (more than 2 categorical variables).

Supplementary Table 5. Patient characteristics for MDACC validation cohort.

	Case	Control	P
Subjects, N	98	361	
age, mean +/-stdev	65 +/- 7.4	63 +/- 8.4	0.035^
BMI, mean +/- stdev	29.2 +/- 4.53	28.9 +/- 4.51	0.748^
PSA Density, mean +/- stdev	0.13 +/- 0.083	0.11 +/- 0.103	0.002^
Testosterone, mean +/- stdev	396 +/- 161.8	396 +/- 162.5	0.922^
Smoking, N (%)			
Yes	61 (62)	206 (57)	0.419^^
No	37 (38)	155 (43)	
5-ARI Treatment, N (%)			
Yes	7 (7)	42 (12)	0.268^^
No	91 (93)	319 (88)	
Statin Use, N (%)			
Yes	52 (53)	165 (46)	0.211^^
No	46 (47)	196 (54)	
Follow-up time (Months), mean (range)	25 (12-96)	41.7 (6-120)	

^ 2-sided Wilcoxon-rank Sum Test

^^ 2-sided Fisher's Exact Test

Supplementary Table 6. Spearman correlation analysis between mRNA levels of CAV1 and mRNA levels of enzymes central to ceramide metabolism in the TCGA Prostate adenocarcinoma gene expression dataset.

TCGA-Prostate Cancer

Pathway	Gene	Spearman Rho	2-sided P
De Novo Pathway	ASAH1	0.07	0.1629
De Novo Pathway	DEGS1	-0.2	0.0001
De Novo Pathway	DEGS2	0.07	0.1684
De Novo Pathway	KDSR	0.4	<0.0001
De Novo Pathway	SPTLC1	0.03	0.5933
De Novo Pathway	SPTLC2	0.03	0.5189
De Novo Pathway	SPTLC3	0.37	<0.0001
Sphingosine Recylcing	ACER1	0.38	<0.0001
Sphingosine Recylcing	ACER2	0	0.9855
Sphingosine Recylcing	ACER3	-0.12	0.0186
Sphingosine Recylcing	CERS1	-0.31	<0.0001
Sphingosine Recylcing	CERS2	-0.32	<0.0001
Sphingosine Recylcing	CERS3	0.38	<0.0001
Sphingosine Recylcing	CERS4	-0.41	<0.0001
Sphingosine Recylcing	CERS5	0.09	0.1009
Sphingosine Recylcing	CERS6	0.01	0.8473
Sphingosine Recylcing	PLPP1 (PPAP2A)	-0.2	0.0001
Sphingosine Recylcing	PLPP2 (PPAP2C)	-0.07	0.1786
Sphingosine Recylcing	PLPP3 (PPAP2B)	0.73	<0.0001
Sphingosine Recylcing	SGPL1	-0.11	0.0336
Sphingosine Recylcing	SGPP1	-0.11	0.0353
Sphingosine Recylcing	SGPP2	0.03	0.5302
Sphingosine Recylcing	SPHK1	0.14	0.0076
Sphingosine Recylcing	SPHK2	-0.27	<0.0001
Sphingomyelinase Pathway	SGMS1	-0.19	0.0004
Sphingomyelinase Pathway	SGMS2	0.09	0.0941
Sphingomyelinase Pathway	SMPD1	-0.16	0.0028
Sphingomyelinase Pathway	SMPD2	-0.29	<0.0001
Sphingomyelinase Pathway	SMPD3	-0.11	0.0336
Sphingomyelinase Pathway	SMPDL3A	0.02	0.7576
Sphingomyelinase Pathway	SMPDL3A	0.02	0.7576
Glycosphingolipid Metabolism	GBA2	-0.29	<0.0001
Glycosphingolipid Metabolism	GLB1	-0.14	0.0074
Glycosphingolipid Metabolism	GALC	0.01	0.8319
Glycosphingolipid Metabolism	GLB1L2	0.09	0.0961
Glycosphingolipid Metabolism	GLB1L	0.1	0.0576
Glycosphingolipid Metabolism	UGCG	0.1	0.0566
Glycosphingolipid Metabolism	GLB1L3	0.18	0.0008
Glycosphingolipid Metabolism	UGT8	0.28	<0.0001
Glycosphingolipid Metabolism	B4GALT5	0.45	<0.0001
Glycosphingolipid Metabolism	B4GALT6	0.45	<0.0001

Supplementary Figure 1. Intra-patient comparison of sphingolipids identified in the Discovery Cohort. Statistical significance was determined by repeated measures 2-sided t-test.

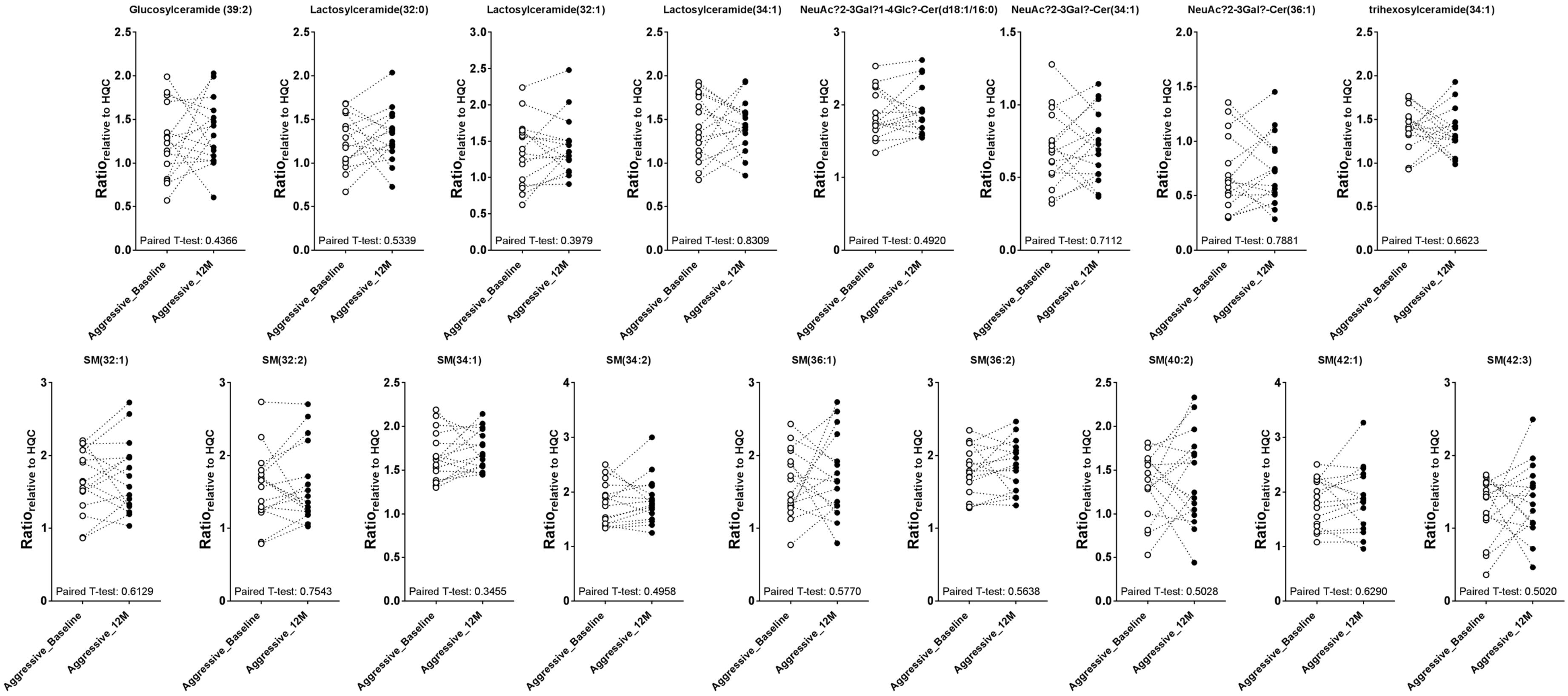

Supplementary Figure 2. Relative lipid composition of SSALPs.

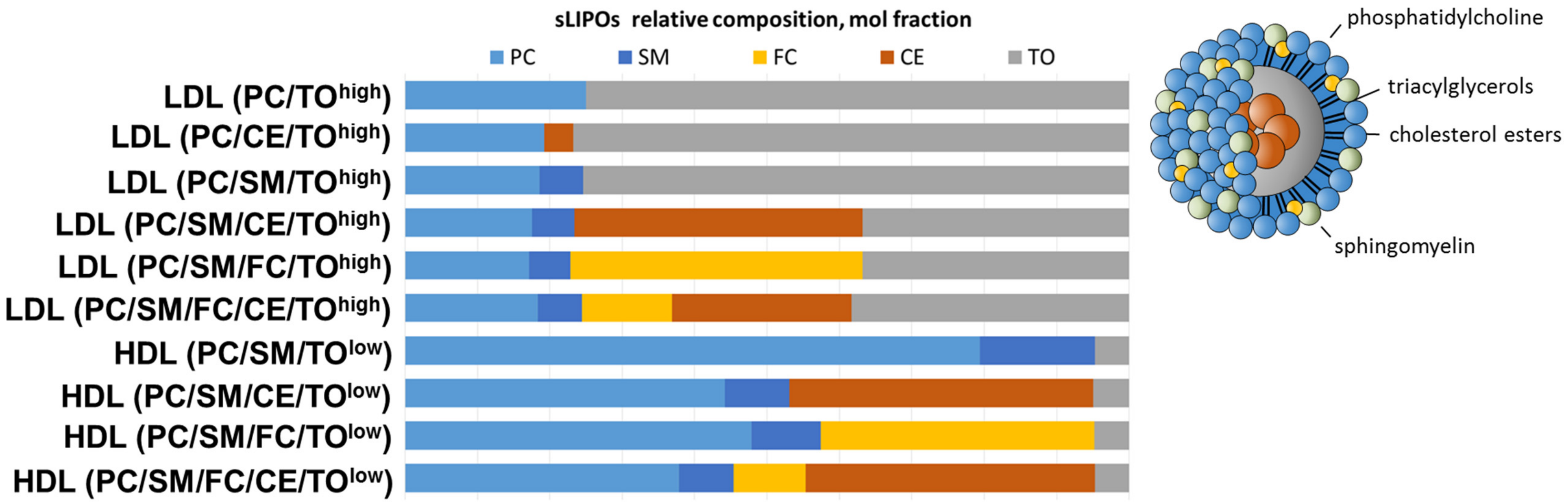

standardized to 6.5 mg/mL total lipids

Supplementary Figure 3. Differences in baseline lipid composition between LNCaP and PC-3M cells. **(a)** Representative immunoblots for Cav-1 in LNCaP and PC-3M prostate cancer cells following Cav-1 overexpression or knockdown, respectively. **(b)** Unsupervised hierarchical clustering heatmap indicating distribution of annotated lipids in LNCaP and PC-3M cells. **(c)** Biochemical network illustrating relationship between altered lipids between LNCaP and PC-3M. Node size and color indicates direction of change (red-increased in PC-3M; green-decreased in PC-3M relative to LNCaP). Edge color and arrows indicate direction of biochemical reaction. Thickened black boarders indicates statistical significance; significance was determined by 2-sided student t-test comparing the aggregate summed intensity of annotated lipids corresponding to the respective lipid domains.

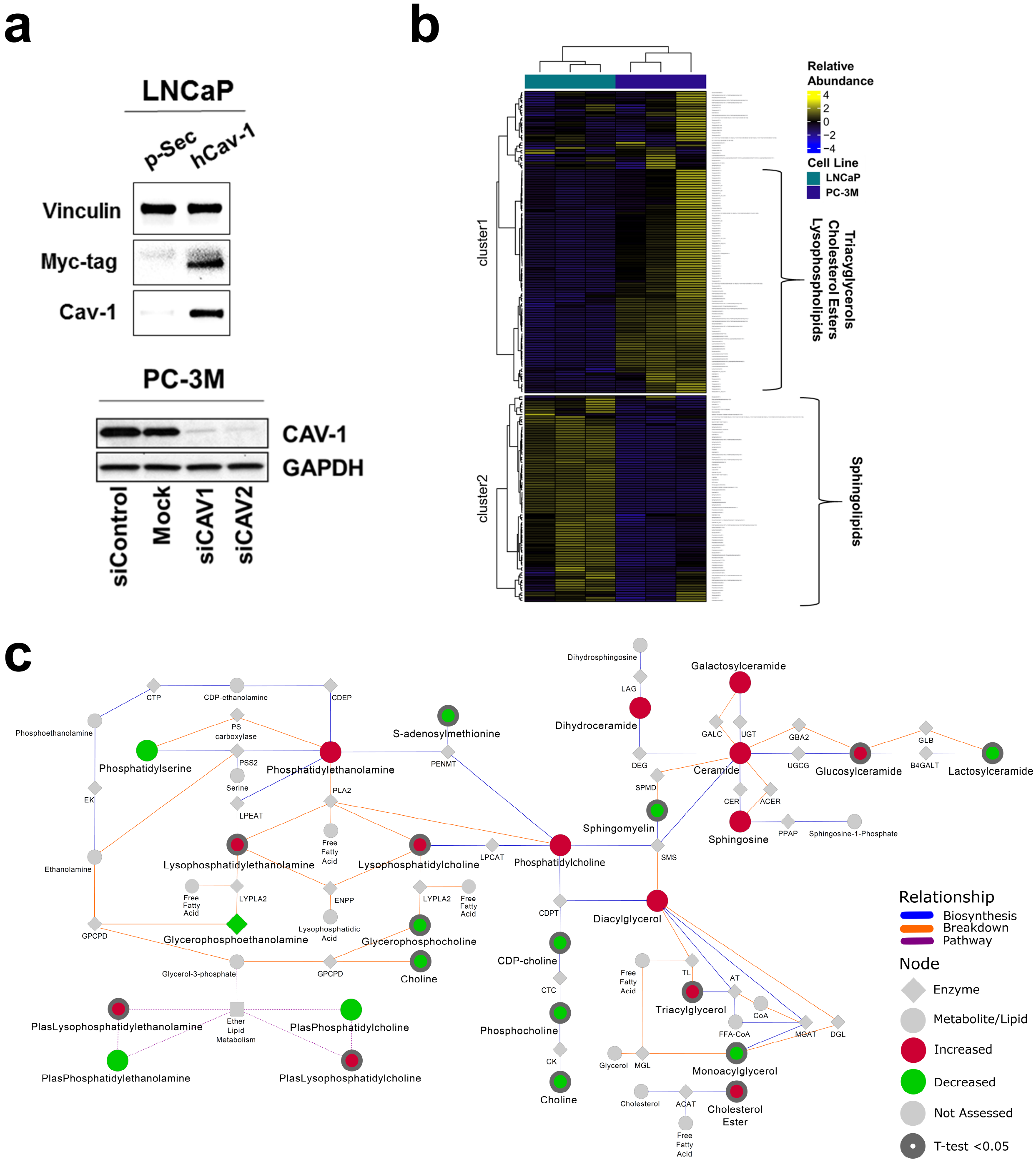

Supplementary Figure 4. Schematic of ceramide biosynthetic pathways.

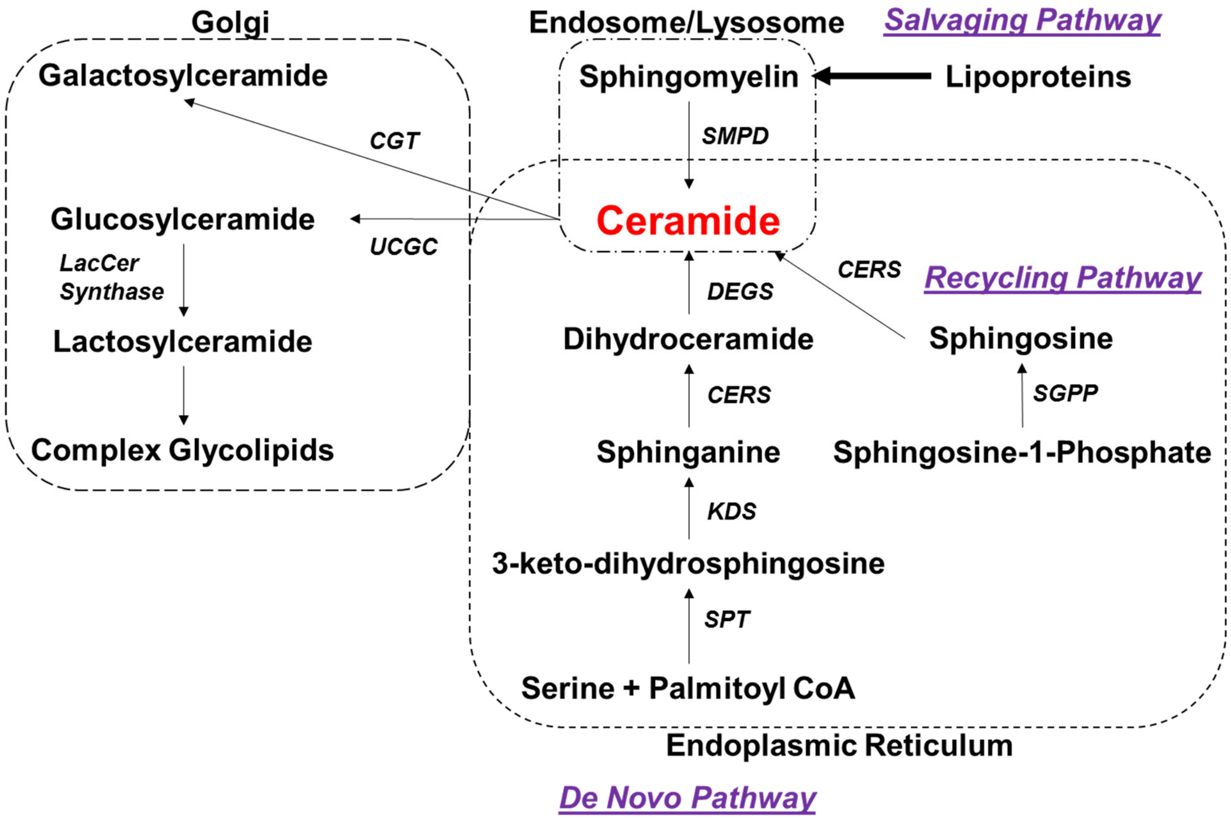

Supplementary Figure 5. Knockdown of *CAV1* in PC-3M prostate cancer cells reduces extracellular sphingomyelin uptake and increases mitochondrial mass and ROS production. **(a)** Violin plots illustrating intensities of TopFluor-SM in PC-3M cells following knockdown of *CAV1*. Statistical significance was determined using One-way ANOVA; pairwise comparisons were performed using Tukey HSD multiple comparison test and adjusted p-value reported. **(b)** Staining for mitochondria mass (MitoTracker Green) in PC-3M cells following knockdown of *CAV1*. Scale bar indicates 20 µm. **(c-d)** Violin plots illustrating intensities of MitoTracker Green **(c)** and MitoTracker CMXRos **(d)** in PC-3M cells following knockdown of *CAV1*. Statistical significance was determined using One-way ANOVA; pairwise comparisons were performed using Tukey HSD multiple comparison test and adjusted p-value reported. Intensity scale bars are provided next to each figure. Digital image acquisition parameters and look up table mappings were uniformly set for all images within each respective panel. Image analysis was performed with NIS Elements software. The entire z-stack was subtracted for background and a threshold was defined based on intensity to select areas containing signal of interest. A mean intensity was calculated for each object (from threshold areas) in all planes for each condition and intensity distributions plotted using violin plots.

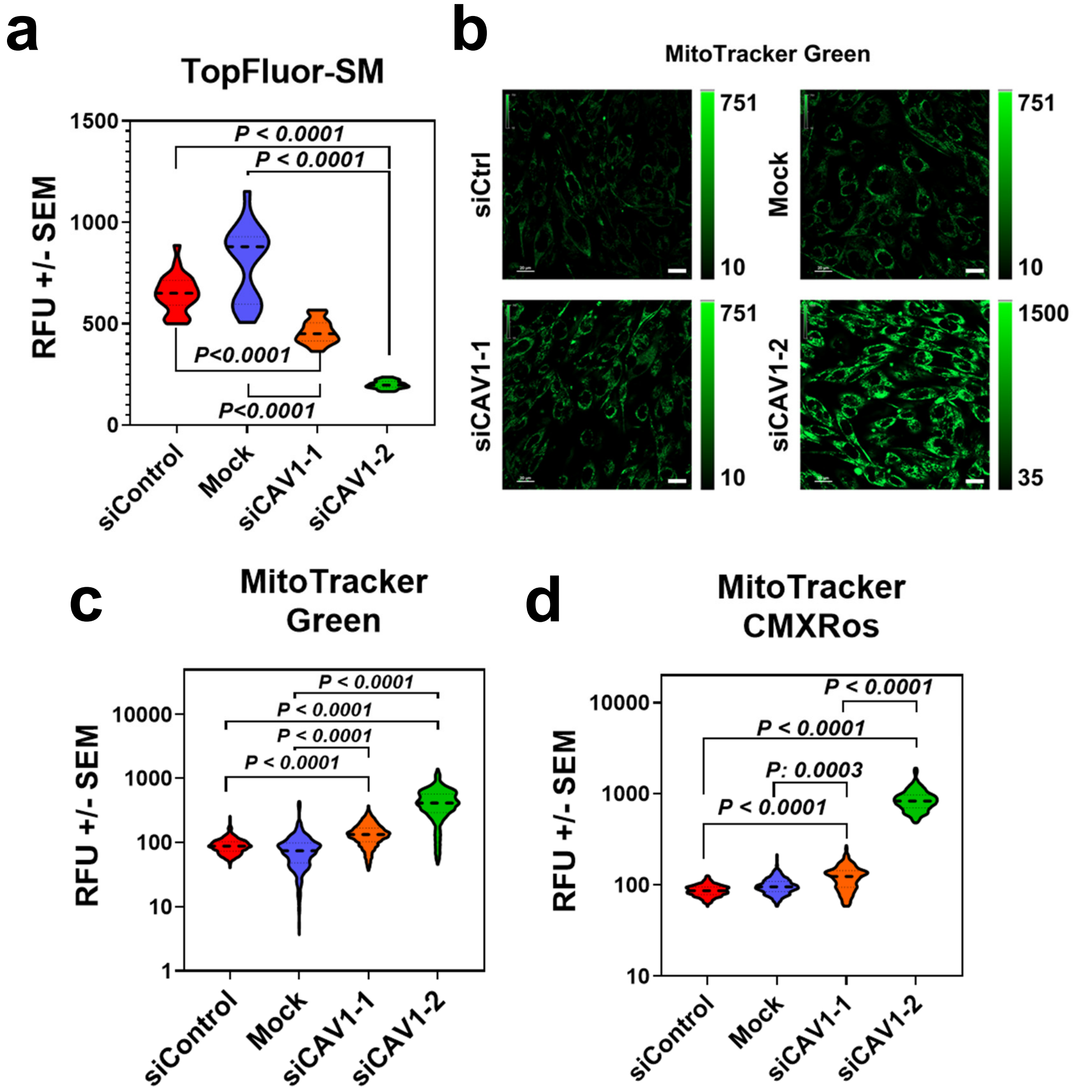

Supplementary Figure 6. Association between extracellular vesicle-associated Cav-1 and density. (a) Baseline levels of Cav-1 in conditioned media from LNCaP, PC-3M and RM-9 prostate cancer cells. **(b)** Schematic of extracellular vesicle fractionation methodology. **(c)** Distribution of Cav-1 in fractionated extracellular vesicles isolated from PC-3M or LNCaP with CAV1 overexpression conditioned media. **(d)** Heatmap illustrating subcellular localization of protein features identified in EVs isolated from conditioned media of LNCaP or PC-3M prostate cancer cells. Subcellular localization is based on the COMPARTMENTS localization evidence database scores. **(e)** Top disease functions and canonical pathways based on Ingenuity pathway analysis of protein features (≥ 5 spectral abundance) identified in PC-3M-derived EVs. # of Molecules indicates the number of protein features identified in PC-3M-derived EVs that correspond to the respective disease function or canonical pathway. Statistical significance determined by 1-sided Fisher's Exact test.

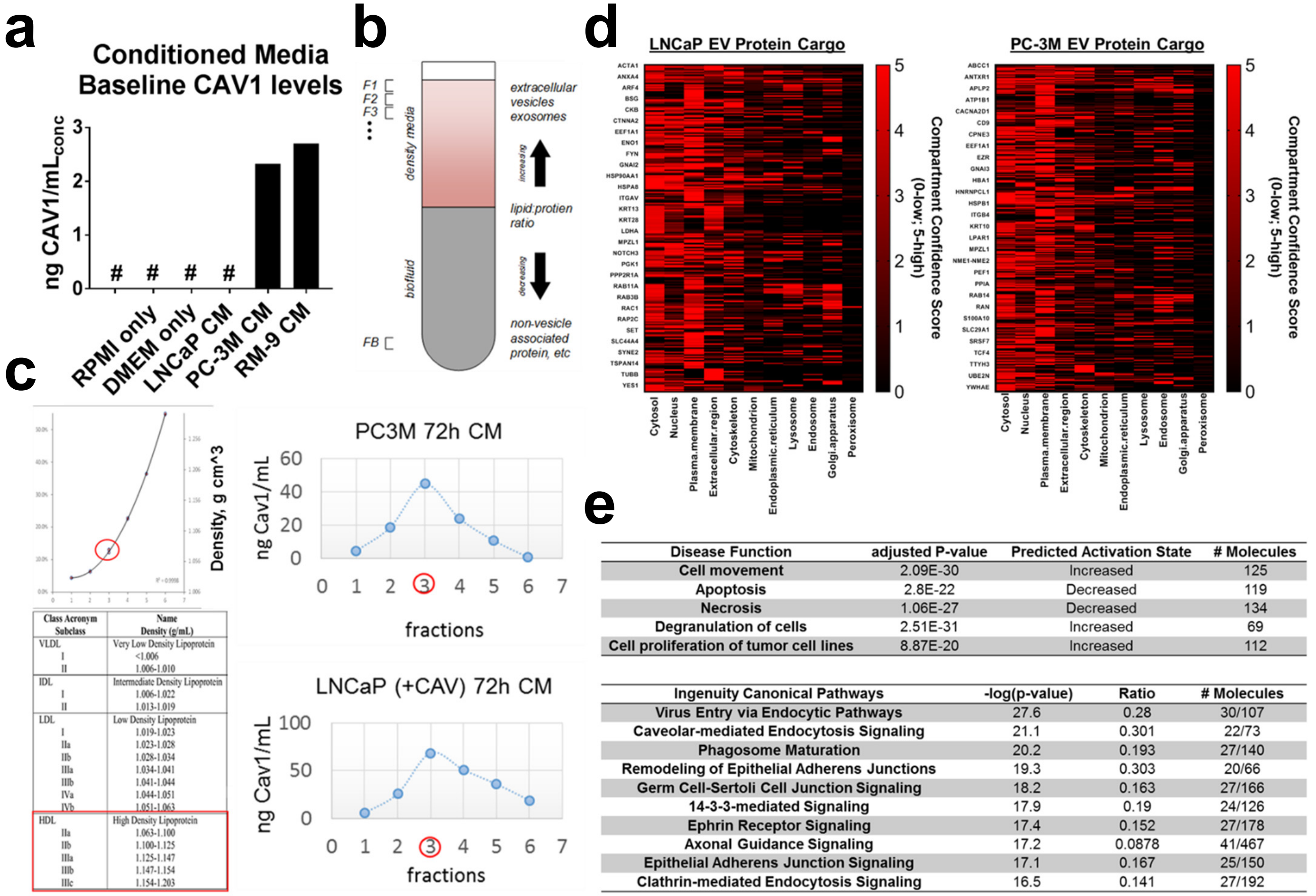

Disease Function	adjusted P-value	Predicted Activation State	# Molecules
Cell movement	2.09E-30	Increased	125
Apoptosis	2.8E-22	Decreased	119
Necrosis	1.06E-27	Decreased	134
Degranulation of cells	2.51E-31	Increased	69
Cell proliferation of tumor cell lines	8.87E-20	Increased	112

Ingenuity Canonical Pathways	-log(p-value)	Ratio	# Molecules
Virus Entry via Endocytic Pathways	27.6	0.28	30/107
Caveolar-mediated Endocytosis Signaling	21.1	0.301	22/73
Phagosome Maturation	20.2	0.193	27/140
Remodeling of Epithelial Adherens Junctions	19.3	0.303	20/66
Germ Cell-Sertoli Cell Junction Signaling	18.2	0.163	27/166
14-3-3-mediated Signaling	17.9	0.19	24/126
Ephrin Receptor Signaling	17.4	0.152	27/178
Axonal Guidance Signaling	17.2	0.0878	41/467
Epithelial Adherens Junction Signaling	17.1	0.167	25/150
Clathrin-mediated Endocytosis Signaling	16.5	0.141	27/192

Supplementary Figure 7. Eliglustat induces cytotoxicity via autophagy/mitophagy. **(a)** Cytotoxicity curves and caspase-3 activation curves for PC-3M prostate cancer cells following challenge with Eliglustat. *N* = 3 biologically independent replicates per experimental condition. Values represent log(relative fluorescence units) +/- StDev. **(b)** Volcano plots illustrating differences in annotated lipid species stratified by lipid domains in PC-3M and RM-9 prostate cancer cells following 6 hr challenge with 128 μM Eliglustat. *N* = 3 biologically independent replicates per experimental condition. Statistical significance was determined by 2-sided student t-test. **(c)** Schematic illustrating lipid catabolism of phospholipid and triacylglycerols.

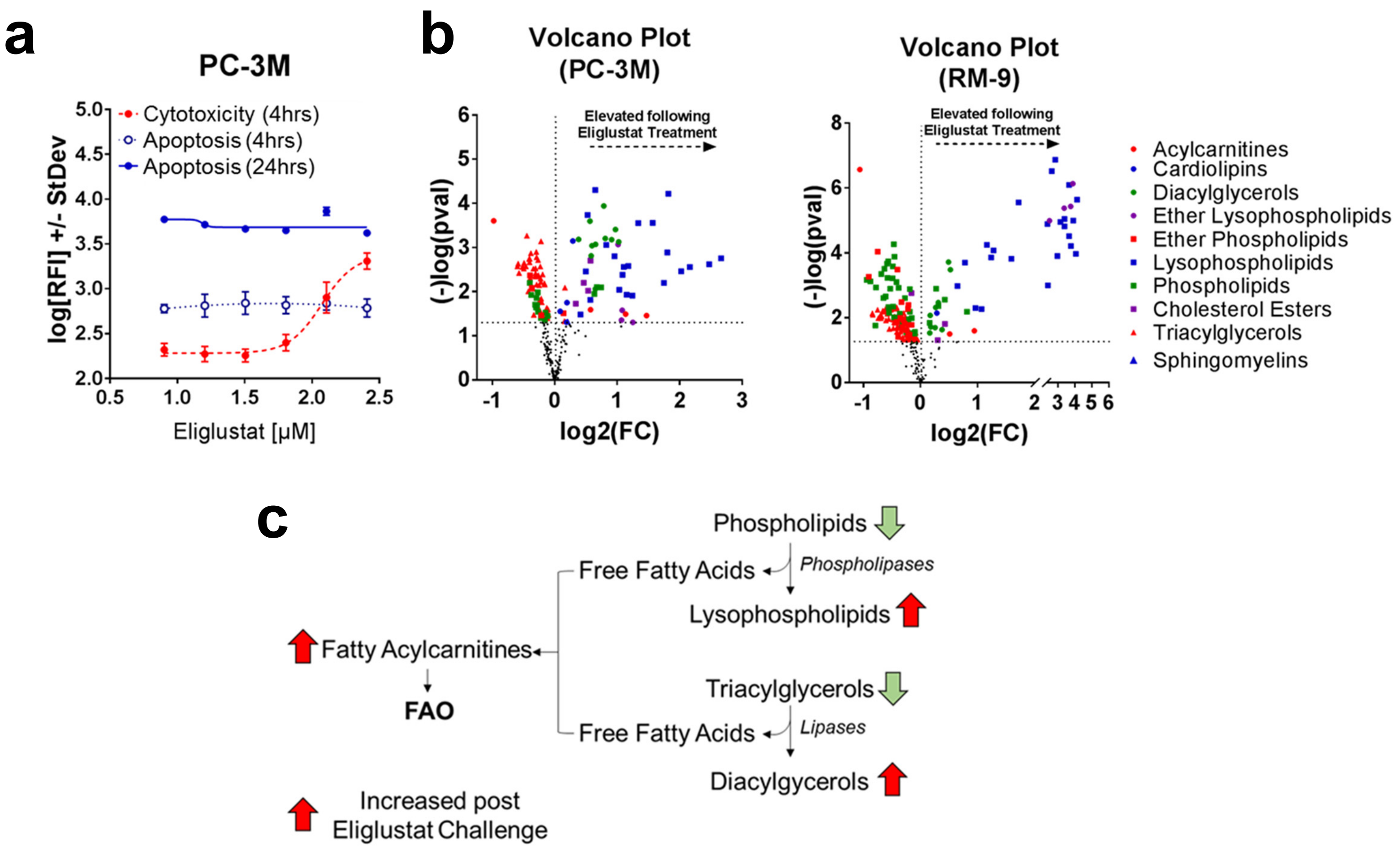

Supplementary Figure 8. Plasma composition of Cav-1, sphingolipid signature and ceramides in RM-9-luciferase bearing B57BL/6N mice following treatment with Eliglustat and abCAV1 alone or in combination. **(a)** Box plots illustrating plasma Cav-1 levels following treatment. The centre line and bottom/upper bounds indicate median and 1st/3rd quartiles, respectively. Whiskers represent minimum and maximum values. *N* = 6 and 3 mice for control mice treated with saline and eliglustat, respectively. *N* = 6 and 7 mice for RM-9 bearing mice treated with saline and eliglustat, respectively. Statistical significance was determined using One-way ANOVA; pairwise comparisons were performed using Tukey HSD multiple comparison test and adjusted *p*-value reported. **(b)** Box plots depicting signature scores for the plasma sphingolipid signature following treatment. ^ only lipids that comprise the lipid signature and that were identified were included. The centre line and bottom/upper bounds indicate median and 1st/3rd quartiles, respectively. Whiskers represent minimum and maximum values. *N* = 10 and 5 mice for control mice treated with saline and eliglustat, respectively. *N* = 19 and 6 mice for RM-9 bearing mice treated with saline and eliglustat, respectively. Statistical significance was determined using One-way ANOVA; pairwise comparisons were performed using Tukey HSD multiple comparison test and adjusted *p*-value reported **(c)** Relative abundance of plasma ceramides +/- StDev following treatment. Statistical significance was determined by 2-sided Wilcoxon-rank sum test comparing the aggregate intensity of annotated ceramides. *N* = 19 and 6 mice for RM-9 bearing mice treated with saline and eliglustat, respectively.

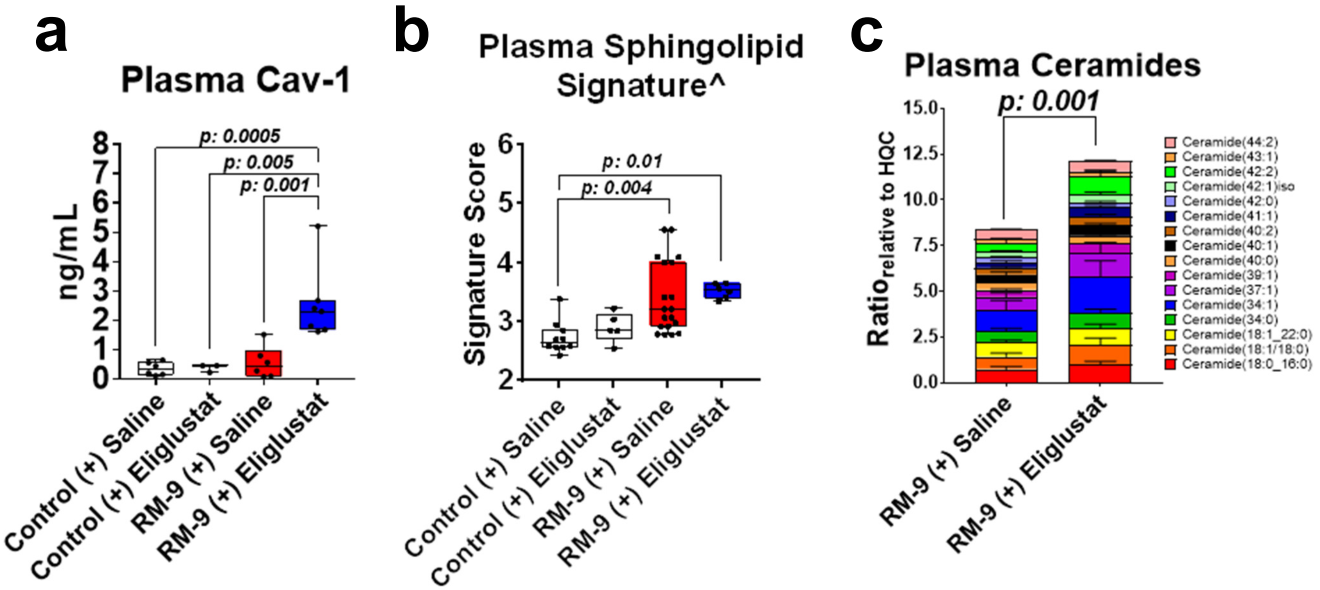

Supplementary Figure 9. Western blot full scans.

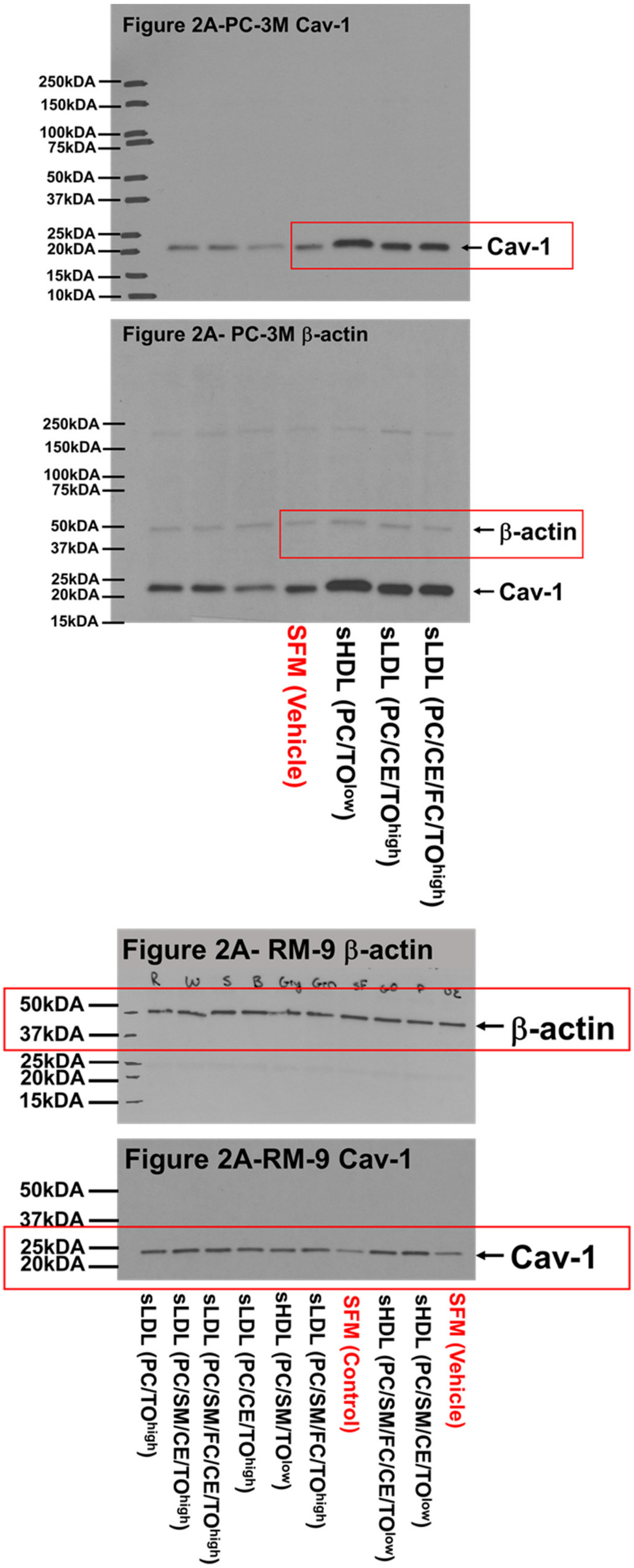

Supplementary Figure 9. Western blot full scans (continued).

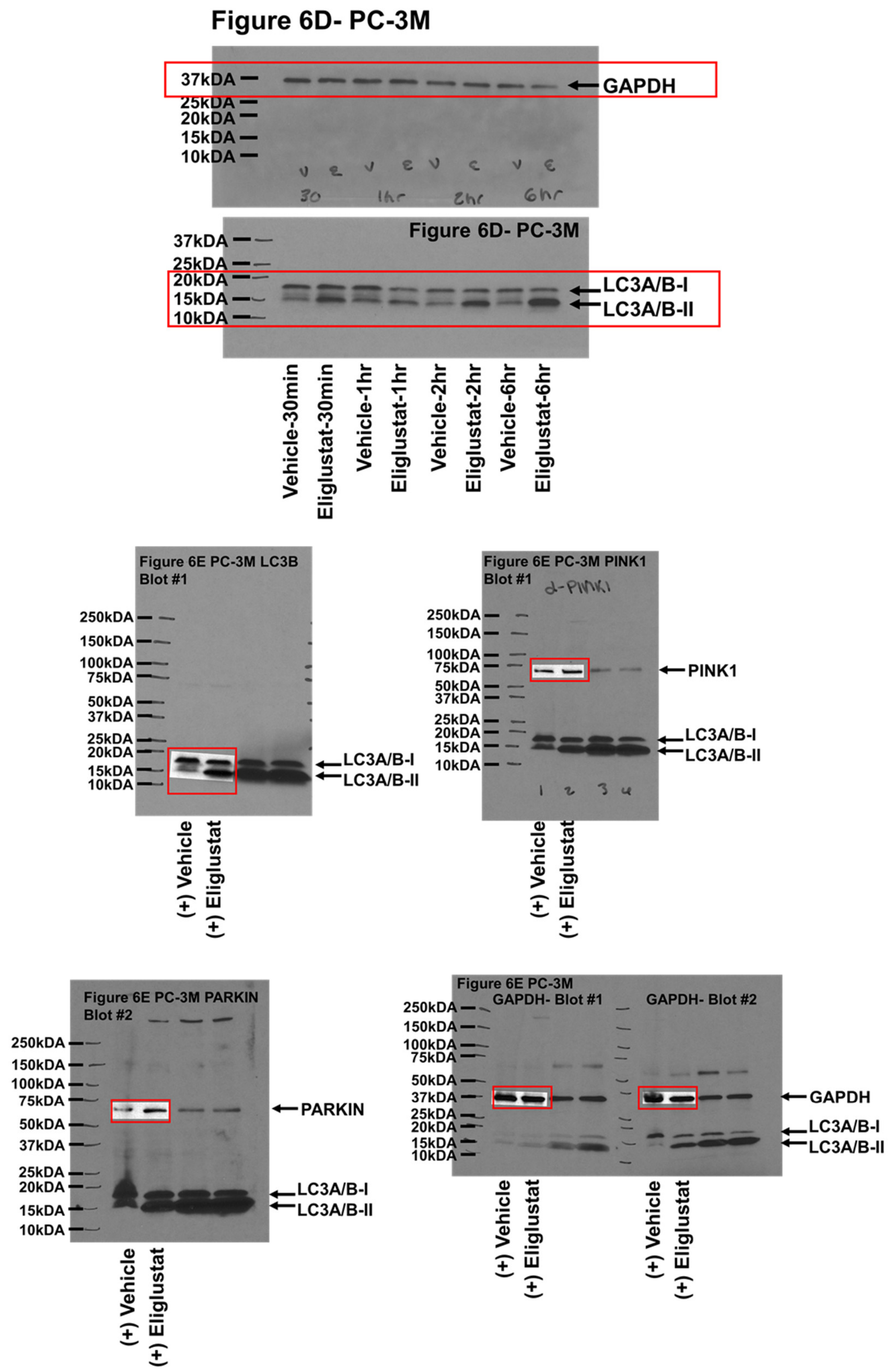

Supplementary Figure 9. Western blot full scans (continued).

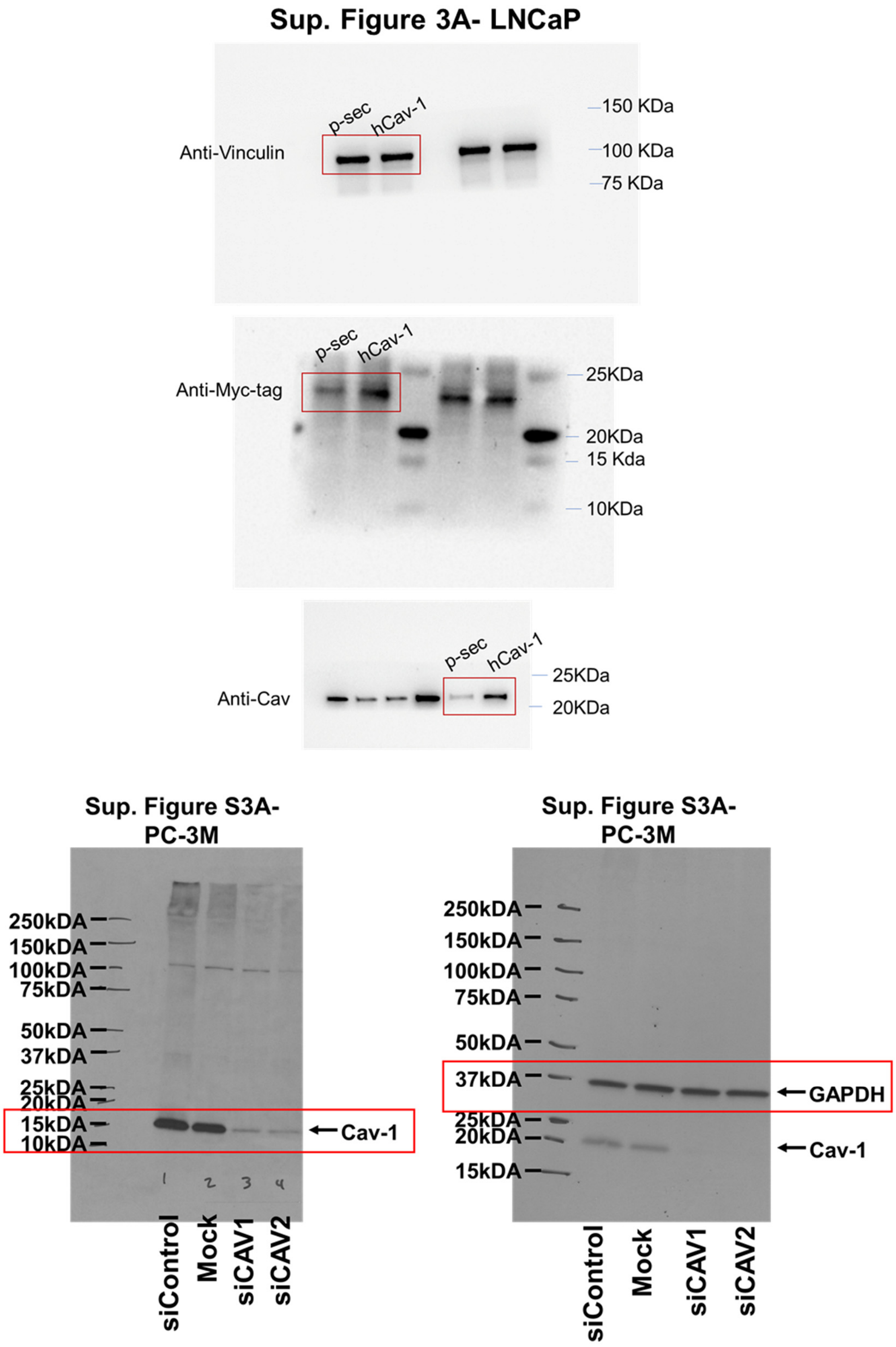

References

1 Vykoukal, J. *et al.* Plasma-derived extracellular vesicle proteins as a source of biomarkers for lung adenocarcinoma. *Oncotarget* **8**, 95466-95480, doi:10.18632/oncotarget.20748 (2017).

2 Capello, M. *et al.* Exosomes harbor B cell targets in pancreatic adenocarcinoma and exert decoy function against complement-mediated cytotoxicity. *Nature communications* **10**, 254, doi:10.1038/s41467-018-08109-6 (2019).

3 Wang, T. *et al.* JAK/STAT3-Regulated Fatty Acid beta-Oxidation Is Critical for Breast Cancer Stem Cell Self-Renewal and Chemoresistance. *Cell metabolism* **27**, 136-150.e135, doi:10.1016/j.cmet.2017.11.001 (2018).

4 Contal, C. and O'Quigley, J. An application of changepoint methods in studying the effect of age on survival in breast cancer. *Computational statistics & data analysis* **30**, 253-270 (1999).
