# Supplementary material for: Caveolin-1-mediated sphingolipid oncometabolism underlies a metabolic vulnerability of prostate cancer
Source: Nat Commun. 2020 Aug 27;11:4279. doi: 10.1038/s41467-020-17645-z (PMC7453025; doi:10.1038/s41467-020-17645-z)
Supplement: Supplementary file 3 — Description of Additional Supplementary Files [file 41467_2020_17645_MOESM3_ESM.docx]

**Description of Additional Supplementary Files**

**Caveolin-1-Mediated Sphingolipid Oncometabolism Underlies a Metabolic Vulnerability of Prostate Cancer**

Jody Vykoukal^1,7,9^, Johannes F. Fahrmann^1,9^, Justin R. Gregg^2^, Zhe Tang^3^,
Spyridon Basourakos^3^, Ehsan Irajizad^5^, Sanghee Park^3^, Guang Yang^3^,
Chad J. Creighton^4,7^, Alia Fleury^1^, Jeffrey Mayo^1^, Adriana Paulucci^6^,
Jennifer B. Dennison^1^, Eunice Murage^1^, Christine B. Petterson^5^, John Davis^2^,
Jeri Kim^3,10^, Samir Hanash^1,7,10^ and Timothy C. Thompson^3,10^

Departments of ^1^Clinical Cancer Prevention, ^2^Urology, ^3^Genitourinary Medical Oncology, ^4^Bioinformatics and Computational Biology, and ^5^Biostatistics and ^6^Genetics, and ^7^McCombs Institute for the Early Detection and Treatment of Cancer, The University of Texas MD Anderson Cancer Center, 1515 Holcombe Boulevard, Houston, TX 77030, USA

^8^Dan L Duncan Comprehensive Cancer Center Division of Biostatistics, Baylor College of Medicine, One Baylor Plaza, Houston, TX 77030 USA

^9^These authors contributed equally: Jody Vykoukal, Johannes F. Fahrmann

^10^These authors jointly supervised this work: Jeri Kim, Samir Hanash, Timothy C. Thompson

**Filename:** Supplementary Data 1

**Description:** Individual normalized gene expression of CAV1 and genes annotated to ontologies related to lipid managing apparti in 333 prostate tumors using TCGA.

**Filename:** Supplementary Data 2

**Description:** Averaged expression of genes annotated to ontologies related to lipid managing apparti in 333 prostate tumors using TCGA.

**Filename:** Supplementary Data 3

**Description:** Lipidomic profiling of prostate cancer cell line-derived EVs.

**Filename:** Supplementary Data 4

**Description:** Lipidomic profiling of RM-9 and PC-3M prostate cancer cells following 6 hour treatment with PDMP, PPMP or eliglustat.
