# Supplementary material for: Caveolin-1-mediated sphingolipid oncometabolism underlies a metabolic vulnerability of prostate cancer
Source: Nat Commun. 2020 Aug 27;11:4279. doi: 10.1038/s41467-020-17645-z (PMC7453025; doi:10.1038/s41467-020-17645-z)
Supplement: Supplementary file 8 — Reporting Summary [file 41467_2020_17645_MOESM8_ESM.pdf]

nature research

Corresponding author(s): Jeri Kim
Samir Hanash
Timothy C. Thompson

Last updated by author(s): Jun 5, 2020

Reporting Summary

Nature Research wishes to improve the reproducibility of the work that we publish. This form provides structure for consistency and transparency in reporting. For further information on Nature Research policies, see Authors & Referees and the Editorial Policy Checklist .

Statistics

For all statistical analyses, confirm that the following items are present in the figure legend, table legend, main text, or Methods section.

n/a	Confirmed	
☐	☒	The exact sample size (*n*) for each experimental group/condition, given as a discrete number and unit of measurement
☐	☒	A statement on whether measurements were taken from distinct samples or whether the same sample was measured repeatedly
☐	☒	The statistical test(s) used AND whether they are one- or two-sided<br>*Only common tests should be described solely by name; describe more complex techniques in the Methods section.*
☐	☒	A description of all covariates tested
☐	☒	A description of any assumptions or corrections, such as tests of normality and adjustment for multiple comparisons
☐	☒	A full description of the statistical parameters including central tendency (e.g. means) or other basic estimates (e.g. regression coefficient) AND variation (e.g. standard deviation) or associated estimates of uncertainty (e.g. confidence intervals)
☐	☒	For null hypothesis testing, the test statistic (e.g. *F*, *t*, *r*) with confidence intervals, effect sizes, degrees of freedom and *P* value noted<br>*Give P values as exact values whenever suitable.*
☒	☐	For Bayesian analysis, information on the choice of priors and Markov chain Monte Carlo settings
☒	☐	For hierarchical and complex designs, identification of the appropriate level for tests and full reporting of outcomes
☐	☒	Estimates of effect sizes (e.g. Cohen's *d*, Pearson's *r*), indicating how they were calculated

Our web collection on statistics for biologists contains articles on many of the points above.

Software and code

Policy information about availability of computer code

Data collection	Gene expression data for prostate cancer cell lines were obtained from Cancer Cell Line Encyclopedia (CCLE) (www.broadinstitute.org/ccle). Gene expression data and clinical data were additionally downloaded from The Cancer Genome Atlas (TCGA) network project webpage (https://tcga-data.nci.nih.gov/tcga/) and the cBioPortal public Data Portal (cbioportal.org). Networks were visualized using cytoscape (https://cytoscape.org/). TCGA prostate adenocarcinoma (PRAD) cases were previously classified by TCGA network using the "iCluster" multi-platform based method as previously described by the Cancer Genome Atlas Research Network.
Data analysis	Statistical analyses were carried out using R statistical software program version 3.6.3 (https://www.r-project.org/) or GraphPad Prism V7 (San Diego, CA)

For manuscripts utilizing custom algorithms or software that are central to the research but not yet described in published literature, software must be made available to editors/reviewers. We strongly encourage code deposition in a community repository (e.g. GitHub). See the Nature Research guidelines for submitting code & software for further information.

Data

Policy information about availability of data

All manuscripts must include a data availability statement. This statement should provide the following information, where applicable:

- Accession codes, unique identifiers, or web links for publicly available datasets
- A list of figures that have associated raw data
- A description of any restrictions on data availability

The MS metabolomics data generated and analyzed during this study have been deposited to the NIH Common Fund's National Metabolomics Data Repository (NMDR), Metabolomics Workbench [https://www.metabolomicsworkbench.org], with Project ID PR00000X accessible via Project DOI: 10.21228/M0000X. The MS proteomics data generated and analyzed during this study have been deposited to the ProteomeXchange Consortium via the PRIDE partner repository with the dataset identifier PXD019441. Gene expression data for prostate cancer cell lines were obtained from Cancer Cell Line Encyclopedia (CCLE) (www.broadinstitute.org/ccle). Gene expression data and clinical data were additionally downloaded from The Cancer Genome Atlas (TCGA) network project webpage (https://tcga-data.nci.nih.gov/tcga/) and the cBioPortal public Data Portal (cbioportal.org). Networks were visualized using cytoscape (https://

cytoscape.org/). TCGA prostate adenocarcinoma (PRAD) cases were previously classified by TCGA network using the "iCluster" multi-platform based method as previously described by the Cancer Genome Atlas Research Network. Other relevant data supporting the findings of this study are available within the Article and Supplementary Information, or are available from the authors upon reasonable request.

Field-specific reporting

Please select the one below that is the best fit for your research. If you are not sure, read the appropriate sections before making your selection.

☒ Life sciences ☐ Behavioural & social sciences ☐ Ecological, evolutionary & environmental sciences

For a reference copy of the document with all sections, see nature.com/documents/nr-reporting-summary-flat.pdf

Life sciences study design

All studies must disclose on these points even when the disclosure is negative.

Sample size	Human biospecimen (plasma) was obtained from patients participating in a prospective clinical cohort that included men diagnosed with localized prostate cancer and that were enrolled on an AS trial protocol between February 2006 and February 2014 (n=825). Of these, 616 patients had at least 1 year follow-up and 491 patients had baseline plasma samples, enabling inclusion in the study. All samples that met inclusion criteria were included in this study.<br>In vitro experiments were performed in biological triplicate unless otherwise stated.<br>For in vivo experiments, subconfluent mouse RM-9 prostate cancer cells, previously transduced with luciferase lentivirus, were injected into 8 week-old C57BL/6N mice (Jackson Labs, Main). A total of 32 mice were used, 9 mice in the treatment arm (60mg/kg of eliglustat) and 23 mice in the control arm (saline).). A sample size of 9 eliglustat (case) and 23 saline control yields >99% power at a significance level (α) of 0.05 to detect a difference in tumor volume of µ_1- µ_2=300 using a one-sided two-sample t-test assuming that tumor volumes are normally distributed with standard deviation of 100 and 150 in eliglustat and saline groups, respectively.
Data exclusions	Of the 825 samples on the AS trial protocol, 334 patients had less than 1 year follow-up or did not have baseline samples available and were therefore excluded from the analyses described in the current study.<br>Individuals were additionally excluded if they presented with active noncutaneous malignancy at any site, had prior radiation therapy for treatment of the primary tumor, or Planned concomitant immunotherapy, hormonal therapy, chemotherapy, or radiation therapy while on protocol.
Replication	Mass spectrometry based analyses were conducted using standardize operating procedures; quality control samples (reference quality control samples as well as batch-specific pooled quality control samples) were included in all analytical runs. Individual human and mouse plasmas were assayed as singlets. Gene expression data was derived from open-source databases (TCGA and CCLE).<br><br>All data generated from in vitro and in vivo replicate experiments are included in the current study. Readouts are reported as mean +/- error (standard deviation or standard error of the mean) unless otherwise specified. In vitro experiments were assessed in biological triplicates unless otherwise stated as to ensure reproducibility. Confocal microscopy images were based on single experiments; however, we note the use of multiple orthogonal techniques and multiple fields to showcase concordance in claims reported herein.
Randomization	Samples analyzed by mass spectrometry were assayed in a blinded randomized fashion. For in vivo experiments, subconfluent mouse RM-9 prostate cells, previously transduced with luciferase lentivirus, were injected subcutaneously into 8 week-old C57BL/6N mice (Jackson Labs, Main). After 3 days, tumor growths were confirmed by bioluminescence and mice were randomly distributed into 2 treatment arms, saline or eliglustat (daily intra-peritoneal injection at 60mg/kg). A separate cohort of non-tumor bearing mice were randomly divided and treated with either saline or eliglustat (daily intra-peritoneal injection at 60mg/kg).<br>Randomization does not apply to in vitro experiments.
Blinding	Samples analyzed by mass spectrometry were assayed in a blinded randomized fashion. For in vivo experiments, a single operator administered either saline or eliglustat via daily IP. Mice were age matched and subjected to the same operating and housing conditions during the course of experiments.

Reporting for specific materials, systems and methods

We require information from authors about some types of materials, experimental systems and methods used in many studies. Here, indicate whether each material, system or method listed is relevant to your study. If you are not sure if a list item applies to your research, read the appropriate section before selecting a response.

Materials & experimental systems

n/a	Involved in the study
	[x] Antibodies
	[x] Eukaryotic cell lines
[x]	Palaeontology
	[x] Animals and other organisms
	[x] Human research participants
	[x] Clinical data

Methods

n/a	Involved in the study
[x]	ChIP-seq
[x]	Flow cytometry
[x]	MRI-based neuroimaging

Antibodies

Antibodies used

α-Cav1 (Cell Signaling, Cat# 3267S), GAPDH (Cell Signaling, Cat# 2118); autophagy marker LC3A/B-I/II (Cell Signaling, Cat# 12741), Parkin (Cell Signaling, Cat# 4211, PINK1 (Cell Signaling#6946), Cav-1 (Santa Cruz sc-894), PCNA (Dako M0879), HMGB1 (Cell Signaling #6893), LC3b (Santa Cruz sc-376604), HRP polymer conjugated secondary antibody (Dako cat#K4061), α-Cav-1 primary antibody (Cell Signaling #3238s), and FITC-conjugated secondary antibody (BD Pharmingen # 554020).

Cav-1mAb was commercially produced by conjugating peptide (LVNRDPKHLNDDVVC) with KLH as immunogen and by immunizing BALB/c mice with the conjugated peptide and then subsequently purified using Protein G affinity column.

Validation

Antibodies purchased from Cell Signaling are produced and validated extensively according to a rigorous protocol and adhere to the hallmarks of antibody validation adapted from Uhlen et al. ("A proposal for Validation of Antibodies.") Nature Methods (2016). Antibodies purchased from Santa Cruz are produced and validated according to protocols established by Santa Cruz Biotechnology, Inc. PCNA (Dako M0879) was validated and certified via SDS-PAGE analysis of immunoprecipitates formed between the antibody and 35S-methionine-labeled extract of CV-1 cells (an immortalized line of monkey kidney epithelial cells) shows reaction primarily with a polypeptide corresponding to PCNA.

Cav-1mAb was commercially produced by conjugating peptide (LVNRDPKHLNDDVVC) with KLH as immunogen and by immunizing BALB/c mice with the conjugated peptide and then subsequently purified using Protein G affinity column as described by Kuo S, et al. HYBRIDOMA (2012).

Eukaryotic cell lines

Policy information about cell lines

Cell line source(s)

LNCaP cell line was obtained from ATCC. PC-3M cell line was a gift from Dr. Isaiah Fidler. RM-9 cells were derived from Zipras/myc9-induced mouse prostate cancer as previously described (Watanabe M et al. Molecular Cancer Research (2009)).

Authentication

PC-3M and LNCaP Cell lines were validated by short tandem repeat DNA fingerprinting with the AmpFℓSTR Identifier kit (Applied Biosystems) in MD Anderson's Cell Line Core Facility. RM-9 is a murine prostate cell line derived from rom Zipras/myc9-induced mouse prostate cancer as previously described (Watanabe M et al. Molecular Cancer Research (2009)).

Mycoplasma contamination

Cell lines were tested and confirmed to be negative for mycoplasma contamination.

Commonly misidentified lines
(See ICLAC register)

No commonly misidentified cell lines were used in the study.

Animals and other organisms

Policy information about studies involving animals; ARRIVE guidelines recommended for reporting animal research

Laboratory animals

8 week-old male C57BL/6N mice were purchased from Jackson Labs.

Wild animals

No wild animals were used in the study.

Field-collected samples

No field collected samples were used in the study.

Ethics oversight

All animal experiments were conducted in accordance with accepted standards of humane animal care approved by MDACC IACUC.

Note that full information on the approval of the study protocol must also be provided in the manuscript.

Human research participants

Policy information about studies involving human research participants

Population characteristics

Human biospecimen (plasma) was obtained from patients participating in a prospective clinical cohort that included men diagnosed with localized prostate cancer and that were enrolled on an AS trial protocol between February 2006 and February 2014 (n=825). Of these, 616 patients had at least 1 year follow-up and 491 patients had baseline plasma samples, enabling inclusion in the study. Sample ages varied, as the study began accrual in 2006 and continued over a ten year time period. The

	primary outcome of interest was time to grade progression, defined as any increase in Gleason score following confirmatory biopsy. Mean age for the patients in whom signature work was done the median was 63.7 +/- std 8.2 years.
Recruitment	Protocol criteria, including surveillance frequency and details regarding upstaging were adhered to as previously described [ref 63]. In summary, with rare exception, patients underwent confirmatory biopsy at study entry. They were then evaluated biannually with digital rectal exam and laboratory studies (serum PSA, testosterone). All biopsies were performed using an 11-core trans-rectal ultrasound-guided scheme. Biopsies were repeated every 1–2 years; if one was negative, then the following year's was omitted. Treatment was discussed with patients who had an increase in tumor volume or Gleason increase, though patients who wished to remain on AS were allowed to do so if approved by their treating physician. Patients were followed until disease progression, treatment, loss to follow-up, elective removal, death or 12/31/2016 (study censor date), whichever came first. EDTA was used in all plasma collections and all specimens underwent a similar number of freeze/thaw cycles prior to obtaining metabolomics data. Plasma was not obtained from fasted individuals.<br><br>In terms of selection bias, the active surveillance study that began in 2006 had relatively broad enrollment criteria, therefore most men who enrolled on AS at MD Anderson were eligible for the study. However, not all men managed on active surveillance at MD Anderson were enrolled on the trial. Therefore, differences between those who do and do not enroll on this study (and whose plasmas were used to study in this analysis) may exist.
Ethics oversight	The surveillance protocol was conducted by a multidisciplinary team of urologists, radiation oncologists and medical oncologists, was approved by the University of Texas MD Anderson Cancer Center Institutional Review Board, and is registered on clinicaltrials.gov (NCT00490763).

Note that full information on the approval of the study protocol must also be provided in the manuscript.

Clinical data

Policy information about clinical studies

All manuscripts should comply with the ICMJEguidelines for publication of clinical research and a completedCONSORT checklist must be included with all submissions.

Clinical trial registration	NCT00490763
Study protocol	Study protocol can be accessed at clinicaltrials.gov (NCT00490763)
Data collection	The goal of this clinical research study is to find out if men who have a type of prostate cancer that has been classified as "low risk" can safely not be treated for the disease. Doctors want to know if patients with "low risk" cancer can avoid or postpone therapy and the related side effects and still live as long as patients who immediately receive therapy. Study began accrual in 2006 and continued over a ten year time period. About 1,000 patients will take part in this study. All will be enrolled at MD Anderson.
Outcomes	The primary outcome of interest was time to grade progression, defined as any increase in Gleason score following confirmatory biopsy. There were no secondary outcomes.
